# Type B Trichothecenes in Cereal Grains and Their Products: Recent Advances on Occurrence, Toxicology, Analysis and Post-Harvest Decontamination Strategies

**DOI:** 10.3390/toxins15020085

**Published:** 2023-01-17

**Authors:** Mohamed A. Gab-Allah, Kihwan Choi, Byungjoo Kim

**Affiliations:** 1 Organic Metrology Group, Division of Chemical and Biological Metrology, Korea Research Institute of Standards and Science, Daejeon 34113, Republic of Korea; 2 Department of Bio-Analytical Science, University of Science and Technology, Daejeon 34113, Republic of Korea; 3 Reference Materials Lab, National Institute of Standards, P.O. Box 136, Giza 12211, Egypt; 4 Graduate School of Analytical Science and Technology, Chungnam National University, Daejeon 34134, Republic of Korea

**Keywords:** type B trichothecenes, deoxynivalenol-3-glucoside, *Fusarium* species, cereals, cereal-based foods, occurrence, toxicology, analytical methods, mitigation strategies

## Abstract

Type B trichothecenes (deoxynivalenol, nivalenol, 3-acetyldeoxynivalenol, 15-acetyldeoxynivalenol) and deoxynivalenol-3-glucoside (DON-3G) are secondary toxic metabolites produced mainly by mycotoxigenic *Fusarium* fungi and have been recognized as natural contaminants in cereals and cereal-based foods. The latest studies have proven the various negative effects of type B trichothecenes on human health. Due to the widespread occurrence of *Fusarium* species, contamination by these mycotoxins has become an important aspect for public health and agro-food systems worldwide. Hence, their monitoring and surveillance in various foods have received a significant deal of attention in recent years. In this review, an up-to-date overview of the occurrence profile of major type B trichothecenes and DON-3G in cereal grains and their toxicological implications are outlined. Furthermore, current trends in analytical methodologies for their determination are overviewed. This review also covers the factors affecting the production of these mycotoxins, as well as the management strategies currently employed to mitigate their contamination in foods. Information presented in this review provides good insight into the progress that has been achieved in the last years for monitoring type B trichothecenes and DON-3G, and also would help the researchers in their further investigations on metabolic pathway analysis and toxicological studies of these *Fusarium* mycotoxins.

## 1. Introduction

Mycotoxins are a diverse group of secondary metabolites produced mainly by toxigenic microscopic fungi, such as *Penicillium*, *Aspergillus*, *Alternaria*, and *Fusarium* species, which can colonize various agricultural commodities in the field site or during storage [1]. The growth of fungal species and subsequent production of mycotoxins are influenced by a complex interaction of biotic and abiotic factors (i.e., fungal interaction, fungal host characteristics, and environmental conditions). Mycotoxins are frequently occurring in both tropical and temperate regions of the world, contaminating food, particularly grains (e.g., wheat, maize, oats, rice, barley, sorghum, and rye), which account for a sizeable proportion of agricultural production. Spices, nuts, coffee, oilseeds, dried peas, and various fruits have been also contaminated with different mycotoxins [2]. Worryingly, mycotoxins are not entirely eliminated throughout food processing operations and can also be detected in processed foodstuffs [3,4]. As reported by the Food and Agriculture Organization (FAO) of the United Nations, over 25% of globally produced cereal crops have been estimated to be contaminated with mycotoxins, thereby resulting in a loss of approximately one billion tons of foodstuffs annually [5,6,7,8]. Mycotoxin-contaminated food and feed can provoke serious acute and chronic health effects conjointly referred to as mycotoxicoses and these effects depend on various factors, such as toxicity level of contaminating mycotoxin, degree of exposure as well as the nutritional status of the individuals [8,9].

**Figure 1:**
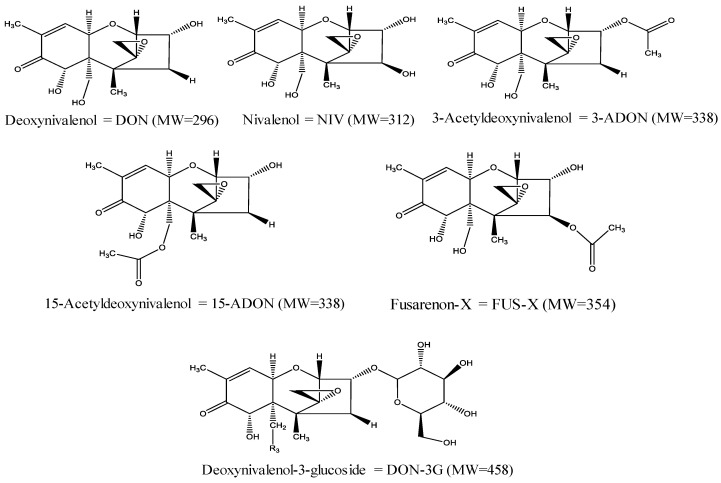
Chemical structures, names, acronyms, and molecular weights of the major type B trichothecenes and deoxynivalenol-3-glucoside.

Over 500 compounds have been identified as mycotoxins up to now, but the most commonly studied mycotoxins with the greatest concern to human and animal health are aflatoxins, zearalenone, trichothecenes, patulin, ochratoxins, and fumonisins [10]. Trichothecenes, a broad spectrum of structurally related compounds, are of significant importance since they are produced by the mycotoxigenic *Fusarium* fungi, which colonize crops at the pre-harvest stages of production [11,12]. Thus, it has become difficult to avoid trichothecene contamination due to the considerable influence of abiotic conditions [13]. These mycotoxins belong to a unique family of cyclic sesquiterpenoids that consist of over 200 analogs with variable toxicological activities [14,15]. Chemically, this family of compounds is characterized by having an olefinic bond with various hydroxyl/acetoxy substitutions and a tetracyclic epoxytrichothene skeleton with a stable C-12, C-13 epoxy group that is responsible for their toxicity [15,16]. They are generally categorized into four subgroups (types A, B, C, and D) on the basis of the characteristics of substituted groups and respective fungal producers [17]. Among the currently available trichothecenes mycotoxins, particular attention has been paid to type B trichothecenes. This group of mycotoxins has been widely researched as they could pose a significant threat to both public health and agro-food systems due to their widespread geographical occurrence [18]. The major forms of type B trichothecenes are deoxynivalenol (DON or vomitoxin), nivalenol (NIV), 3-acetyldeoxynivalenol (3-ADON), 15-acetyldeoxynivalenol (15-ADON), and fusarenon-X (FUS-X; 4-acetylnivalenol) [18,19]. As can be noted from Figure 1, these mycotoxins share a common non-macrocyclic structure and a keto (carbonyl) function at position C-8. They are generally produced by *F. culmorum* and *F. graminearum* fungi species, which commonly contaminate a wide range of cereal grains including wheat, corn, oats, barley, and rice [18,19].

Interestingly, living plants can bind certain mycotoxins with different polar moieties, such as glucose, sugar, amino acid, sulfate, etc., via an enzymatic reaction in plant phase II metabolism to transform mycotoxins into less toxic metabolites (i.e., masked or conjugated mycotoxins) [15,19,20]. In recent times, biologically modified mycotoxins have raised substantial concerns relating to the safety of contaminated food commodities as these compounds are more polar than the precursor mycotoxins and normally elude routine determination due to unusual physicochemical properties [1,20]. The most prevalent masked mycotoxin of DON is DON-3G, which was originally isolated and identified from *Zea mays* cell suspension cultures treated with DON [20]. In the 1980s, DON-3G was first described as a plant conjugate of DON. A potential health hazard of DON-3G for consumers is arising from the possible hydrolysis and reactivation of its toxic-free form (DON) at specific conditions in mammal metabolism [19,20,21]. So far, the natural occurrence of DON-3G was first reported in naturally incurred maize and wheat samples in 2005 [20]. Since then, it has been detected in different cereal grains, animal feeds, and cereal-based products, such as beer and malt [19,22,23,24,25]. The chemical structure of DON-3G is depicted in Figure 1.

Given the widespread distribution of DON and other type B trichothecenes, the ingestion of contaminated cereal products appears to constitute a major source of their exposure in humans [18,26]. Furthermore, DON in feeds is one of the major mycotoxins that has been associated with significant economic losses due to its contribution to the reduced performance in livestock productivity [27]. To actively implement efficient scientific control strategies for reducing the exposure of humans and animals to type B trichothecenes, surveillance and monitoring studies using reliable analytical methodologies are of paramount significance to investigate the real incidence and dietary intakes of these mycotoxins in raw food materials and their final products. This review provides an overview of the toxicology-related aspects of major type B trichothecenes and their occurrence in a variety of foods. Additionally, different methodological approaches and detection techniques proposed for their determination, as well as management and decontamination strategies for limiting their presence in foods are described.

## 2. Factors Affecting Type B Trichothecenes Production

**Figure 2:**
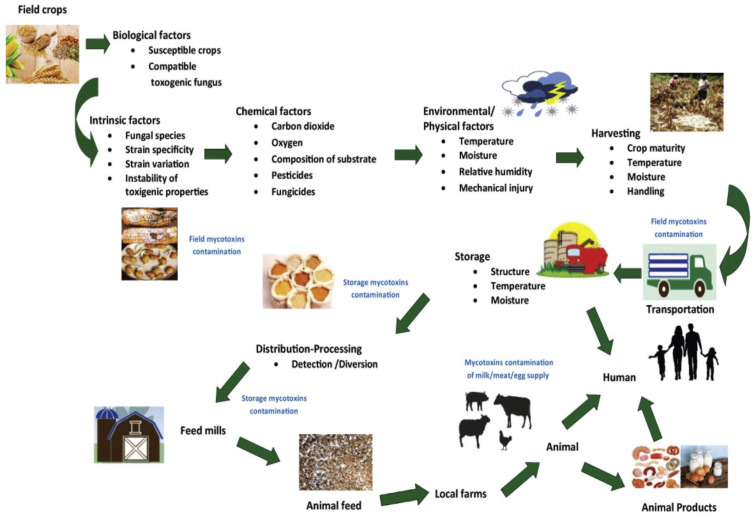
Factors influencing mycotoxin production in the food chain. Reproduced with permission from Haque et al. [10]. Copyright 2020, Elsevier.

The growth of fungal species and subsequent production of mycotoxins at different stages of crop production are influenced by a complex interaction of biotic and abiotic conditions. Figure 2 shows the conditions that influence mycotoxins production in food and feed chains [10]. These conditions are mainly related to environmental/physical, biological, and chemical factors. The environmental factors involve temperature (0–50 °C), relative humidity of surroundings (70%), precipitation patterns, water activity (>0.88) “the amount of free water available in food that can be utilized by microorganisms”, and mechanical injury [10,28]. These climatic conditions, especially moisture content and ambient temperature, are extremely important factors in determining the occurrence of mycotoxigenic fungi, their level of colonization, and subsequent accumulation of relevant mycotoxins in the field [29,30,31]. Thus, variations in fungal development and mycotoxin generation are evident across geographical locations owing to variances in climatic conditions and fungal growth requirements [32].

Mycotoxigenic fungi are conventionally classified as “field” (also known as plant-pathogenic) and “storage” (also known as saprophytic/spoilage) species [33,34]. Field fungi attack seeds while the plant crops are growing in the field and require high moisture levels (≥20%) to thrive. These fungi species involve *Fusarium*, *Cladosporium*, *Claviceps*, *Helminthosporium*, *Neoitphodium*, *Gibberella*, *Cladosporium*, and *Alternaria* [34]. Storage fungi attack seeds or grains while they are being stored and require lesser moisture content (13–18%) than field fungi. Thus, they are not associated with serious food safety issues at the pre-harvest stages. Moreover, they can grow at equilibrium moisture contents with relative humidity levels of 70–90% in the absence of free water [34]. *Aspergillus* and *Penicillium* are among the fungal species that belong to this group. However, it is widely accepted that the majority of contaminations in storage facilities are caused by infections that previously originated in the field [34]. Generally, no further deterioration in grains will take place if the product is dry and stored in a dry location. Nonetheless, if there is water leakage, condensation, or insect/rodent activity, mycotoxin-causing fungus growth will develop.

Chemical factors that influence mold growth and mycotoxin synthesis include fungicides/pesticides application, oxygen, and carbon dioxide concentrations, as well as the composition of the substrate. Meanwhile, other biological factors are related to susceptible crops (substrate), compatible toxigenic fungi (fungal strains), strain specificity, variation, and instability [8,10]. Under field conditions, a plant’s susceptibility to infestation and colonization by toxigenic fungi is often enhanced by stress and subsequent reduction in vitality. Certain fungal strains are capable of creating many mycotoxins, while a single mycotoxin may be generated by multiple fungi [35]. Molds may be found on a variety of substrates, i.e., nearly every kind of food could be infected by molds since the nutrients (carbon and nitrogen) necessary for their development are available in food, particularly those that are high in carbohydrates [36]. However, the exact reason why fungi prevail on a particular food item remains unknown. During storage of grains, the growth of fungal species and subsequent production of mycotoxins arise generally from multiple factors interacting with one another, including moisture content, temperature, fungal abundance, oxygen (O_2_), and carbon dioxide (CO_2_) concentration, pH of the substrate and its composition, mechanical damage, and microbial interactions [34,37].

## 3. Toxicity of Type B Trichothecenes

Type B trichothecenes are considered the most widespread group of *Fusarium*-generated mycotoxins, which are typically characterized by containing a tetracyclic 12, 13-epoxytrichothene skeleton, accounting for many of their toxicological effects [18]. Type B trichothecenes are readily absorbed via different routes. The main route for potential exposure to these mycotoxins is through dietary ingestion of contaminated foods and feeds, but inhalation of toxigenic spores and direct skin contact have also been identified as possible sources of infection [38]. Trichothecenes are very stable mycotoxins; they are not eliminated completely from cereal-based foods during milling processing and are not degraded entirely by high temperatures [39,40]. Therefore, they are commonly detected in grains as well as their processed products, such as flour, baking products, breakfast cereals, pasta, malt, and beer [14,23,24,25,40,41,42]. Trichothecenes are harmful to plants, humans, farm animals, fish, birds, and various eukaryotic species in general. The cytotoxic potency of these mycotoxins is variable depending on the certain contaminating toxin and the studied animal species. An acute intoxication via oral, dermal, or respiratory exposures induces a spectrum of adverse effects, including hemorrhage, leukopenia, hematological disorders, abdominal pain, growth retardation, circulatory shock, immunosuppressive effects, and toxicity of the central nervous system, leading to the loss of appetite, nausea, fatigue, fever, and vomiting (emesis) [9,14,18,24].

DON and other type B trichothecenes are seriously intoxicating in view of their further capability to be topically absorbed, and their metabolites affect the skin, gastrointestinal tract, liver, kidney, immune, and hematopoietic progenitor cellular systems [43]. Once these mycotoxins disseminate into the systemic circulation, they readily impact tissue proliferation. Exposure of farm animals to feed containing moderate levels of DON leads to temporary feed refusal and a reduction in weight gain. The susceptibility to DON was shown to be varying among animal species in the following decreasing order: “pigs > mice > rats > poultry ≈ ruminants” [44].

The main targets of type B trichothecenes are leucocytes; they have the potential to up- and downregulate immunological response by interrupting intracellular signaling inside leukocytes [45]. Based on their dose, incidence, and duration of exposure, they typically induce immunosuppressive effects or act as immunostimulatory. Type B trichothecenes demonstrate their chronic toxicity through binding to the 60S subunit of ribosomes as a consequence of interaction with peptidyl transferase enzyme, thereby rapidly activating mitogen-activated protein kinases (MAPKs). This typically causes inhibition of peptide bond formation and suppression of DNA biosynthesis in eukaryotes. Furthermore, these compounds can inhibit mitochondrial function and reduce cell proliferation [46,47].

DON has been shown to induce apoptosis and dysfunction in mouse kidneys and diverse immune cells, such as dendritic cells, macrophages, and lymphocytes via the response to ribotoxic stress in a process termed a “ribotoxic stress syndrome” [48]. Pharmacological studies have indicated that two important upstream transducers of DON-induced MAPKs, namely double-stranded RNA (dsRNA)-activated protein kinase (PKR) and hematopoietic cell kinase (Hck) contribute to the activation of MAPKs, thus increasing DON-induced gene expression and apoptosis [49]. DON can also increase the susceptibility of farm animals to porcine reproductive and respiratory syndrome virus (PRRSV) infections by influencing the specific humoral immune responses, thereby enhancing the synergistic effects of the toxin and viral diseases on weight gain, lung lesions and mortality [50,51].

Recently, intestinal epithelial cells have also been identified as one of the main sensitive targets for these mycotoxins [52]. DON and NIV were shown to induce cell necrosis in intestinal epithelial cells and alter their capacity to proliferate. In former studies, it was demonstrated that a DON dose of 1–7 mg/kg diet can dramatically reduce the absorption area of the villus surface and also can increase intestinal permeability, resulting in low viability and immunological function in poultry [53]. On the same track, it has been proven that DON can impact the intestinal epithelium in pigs. It also modulates the barrier function of the intestinal epithelial cells (IECs) by altering the tight junction proteins (TJP) and mRNA expression, thereby affecting absorption and nutrient intake by enterocytes [54]. Consequently, researchers believe that trichothecenes can promote a variety of chronic intestinal inflammatory disorders, such as inflammatory bowel disease, and also contribute to food-related allergies, especially in youngsters [52].

NIV and FUS-X are more hazardous to humans and domestic animals compared to DON [13,48]. Recently, the ingestion of NIV-contaminated food from certain locations in China has been linked to an increased incidence of esophageal and gastric carcinomas [55]. During in vitro microsomal studies, FUS-X was almost completely (>90%) converted to NIV in the liver and kidney. FUS-X mainly affects organs that have rapidly growing cells such as the gastrointestinal tract, spleen, hematopoietic tissues, and the thymus, thereby eliciting intestinal inflammation and apoptosis. Moreover, genetic material alteration, which typically causes chromosomal aberrations (CAs), cell cycle delays, and sister chromatid exchanges, are toxicological issues related to FUS-X. In mice, NIV and FUS-X induced much longer, persistent anorectic effects than DON and other acetylated derivatives in the rank order of NIV > FUS-X > DON ≈ 3-ADON ≈ 15-ADON for IP exposure and FUS-X > NIV > DON ≈ 3-ADON ≈ 15-ADON for oral exposure [56]. In another study on mice and mink, FUS-X and NIV demonstrated longer durations of emesis than DON, which is more likely due to their slower elimination rates [57]. They are also potent inhibitors of protein, RNA, and DNA synthesis in mammalian cells [58]. Their exposure to poultry has been associated with certain negative impacts, including decreased gizzard weight and erosions in chickens, along with paler and more fragile than normal livers and kidneys in egg-laying hens [59,60,61]. Other critical toxicological effects in mice are related to intrauterine growth retardation, immunotoxicity, myelotoxicity, and hematotoxicity [62]. Despite all the recent information on trichothecenes’ toxicity, evidence of carcinogenicity from human and animal studies is still inadequate. Therefore, the International Agency for Research on Cancer (IARC) has classified certain trichothecenes (DON, NIV, and FUS-X) as IARC Group 3 compounds, which means that they are not proven to be carcinogenic to humans [63].

Living plants and fungal metabolism can modify the structures of DON through a certain defense mechanism, resulting in the natural occurrence of DON acetylated derivatives, such as 3- and 15-acetyldeoxynivalenol (3-ADON and 15-ADON), and modified (i.e., masked) forms such as deoxynivalenol-3-glucoside (DON-3G), which are of particular relevance in contaminated food and feed [48,64]. Contamination levels of the acetylated forms of DON can reach 10–20% of the total DON concentration and their occurrence has been studied in *Fusarium*-contaminated cereals from different countries [18,19,24,26]. Toxicological effects posed by ADONs in humans and animals are similar or even greater than that of DON due to their rapid absorption into the intestine [65]. The toxicological significance of DON acetylated derivatives has been recently highlighted in various reports for mammals with respect to the immune system, intestinal issues, cell cycle, and oxidative stress [48,66,67,68,69]. Exposure of human U937 macrophages to 3-ADON and 15-ADON can induce inflammatory cytokines, similar to DON [70]. In human in vitro cells, 3-ADON induced inhibition to the proliferative response of peripheral lymphocytes and reduced their capacity to generate antibodies, without modulating their viability [71]. In mice, 15-ADON and DON showed comparable effects in respect of reduced feed consumption and body weight gain [72]. Pinton et al. [65] explored the toxicological effects of DON, 3-ADON, and 15-ADON on the intestine of piglets regarding cell proliferation, barrier function, and intestinal structure. A general finding was that these mycotoxins demonstrate distinct cytotoxicity on the piglet intestine with respect to histological lesions, paracellular permeability, and cell proliferation ranked in the order as follows: 15-ADON ≫ DON > 3-ADON [65]. Even at a lower dosage than 3-ADON and DON, 15-ADON has a greater effect on decreasing the expression of the tight junction proteins, and activation of the MAPK (ERK1/2, p38, and JNK) in differentiated intestinal epithelial cells, explants, and jejunum of exposed piglets [65]. Similar findings were observed in human Caco-2 cells [73,74]. In other reports on human and porcine intestinal cells, 3-ADON was twofold less toxic in inducing cell proliferation than 15-ADON [75].

The main concern over the presence of conjugated or masked mycotoxins (i.e., DON-3G) in contaminated food and feed stems from the fact that their unusual physicochemical properties can present challenges for routine determination while the structures are still maintaining toxic effects. As conjugation is a detoxification process in plants against the effects of xenobiotic compounds such as mycotoxins, DON-3G appears to be less harmful compared to its parent form [52,76]. According to in vitro data, DON-3G is stable against acidic conditions in the stomach and is improbable to be hydrolyzed by the action of digestive enzymes [21]. Due to its reduced potential to evoke gut satiety hormones and anorectic responses, DON-3G is less potent than DON to stimulate emesis in mice [77]. DON-3G is also ineffective in inducing pro-inflammatory cytokine after oral exposure in mice, and incapable to stimulate ribotoxic stress in porcine intestinal epithelial cells [78]. This may be attributed to the steric hindrance effect of the glucose molecule, which suppresses the interaction with the A-site binding pocket at the peptidyl transferase center (PTC) of the ribosome [48]. Furthermore, DON-3G exhibited substantially smaller cytotoxicity compared with DON in treated Caco-2 cells derived from the human colon, porcine intestinal epithelial cells (IPEC-J2), and intestinal explants [79].

Several previous reports indicated that DON-3G can be partly hydrolyzed in the body under the influence of bacterial metabolism in the intestines, thereby increasing the bioavailability of the precursor toxin (DON) [21,41,80]. Therefore, clinical observations suggested that the modified mycotoxins could be responsible for the unpredicted higher degree of mycotoxicosis in animals that is not correlated with mycotoxins levels identified in the corresponding diet [81]. In this sense, DON-3G was considered as a possible factor involved in the total DON-induced toxicity according to the Joint FAO/WHO Expert Committee on Food Additives (JECFA) [82].

Based on the scientific literature, type B trichothecenes and D3G have shown a high incidence of co-contamination in cereal grains since it has been commonly established that *Fusarium* fungi can produce more than one mycotoxin within the same commodity [26,83,84]. According to the abovementioned toxicological effects of type B trichothecenes and based on the food safety point of view, the co-occurrence of these mycotoxins across cereal grains raises a major concern in public health. This is mainly because the current guidelines and risk assessments were typically established based on the toxicity studies of each individual mycotoxin, whereas the interaction among multiple mycotoxins in foods or diet may result in various impacts on human health, and therefore would increase the toxicological hazard due to synergistic effects of possible combined exposure [24,85].

## 4. Legislation of Type B Trichothecenes in Foodstuffs

As stated earlier, type B trichothecenes are considered the most widespread group of *Fusarium*-generated mycotoxins, which commonly infect a wide range of food commodities [18,26]. Since these mycotoxins can be formed at the pre-harvest stages of crop production, their incidence in agricultural crops is highly dependent on environmental conditions and is sometimes inevitable [18]. Due to the potential health hazards of these mycotoxins and the tremendous economic impacts arising from the losses in food quality, comprehensive food safety regulations regarding the maximum levels of these mycotoxins are essential to actively avoid their serious effects and to protect consumer health. Among the currently available type B trichothecenes, DON is the only regulated mycotoxin, while the current regulations do not specify maximum levels of other type B trichothecenes in foods. Although many developing countries still have no specific DON regulations for food commodities, they have recognized that minimizing mycotoxin contamination will not only reduce the financial burden on health care but will also provide international economic benefits such as increased exports to the lucrative European markets. Table 1 lists the maximum regulatory limits of DON in different foodstuffs fixed by various regulatory authorities, including the US Food and Drug Administration, European Union, and Food and Agricultural Organization of the United States/Codex Alimentarius, as well as some Asian countries [86,87]. In a practical situation of the ecological system, DON is more likely to be found in food commodities with other mycotoxins including its modified and masked forms. This could explain the variation in the maximum regulatory limits based on geographical origins and types of food commodities which creates new challenges for regulatory agencies responsible for establishing regulations, scientific legislation, and standards [88].

It is also important to note that the Scientific Committee on Food (SCF) in 2002 proposed tolerable daily intakes (TDIs) for NIV and DON at 0.7 and 1 µg/kg body weight, respectively [89]. However, there is still increasing evidence that 3-ADON and 15-ADON can contribute to total DON-induced toxicity due to their deacetylation during mammalian digestion [66]. Therefore, in 2010 the Joint FAO/WHO (World Health Organization) Expert Committee on Food Additives (JECFA) extended the previous provisional maximum tolerable daily intake (PMTDI) for DON (1 μg/kg body weight) to a group of DON and its acetylated derivatives and also defined a group acute reference dose (ARfD) at 8 μg/kg body weight, based on investigations on mice that showed a reduced feed consumption and growth retardation [82,90]. However, DON-3G was not included in the group PMTDI since the aspect regarding toxicological relevance and occurrence was still being considered. In 2017, with increasing JECFA evaluation and available toxicological data from human intoxications from Asian countries, the European Food Safety Authority (EFSA) CONTAM Panel reported on the risks to human and animal health related to the presence of DON, its acetylated derivatives and modified forms in food and feed [91]. In this report, EFSA suggested that the risk assessment needs to account for DON, including its acetylated derivatives and modified form as additional contributing factors to dietary exposure to DON, and therefore used the same group PMTDI (1 µg/kg body weight per day), but for the sum of DON, 3-ADON, 15-ADON, and DON-3G in infants, toddlers, and other children, and applied it for the overall human risk assessment [43,91].

**Table 1:** Maximum regulatory limits of DON in different foodstuffs established by various regulatory authorities.

Regulation Authority	Foodstuff	Maximum Level μg/kg	Reference
European Commission	Unprocessed durum wheat, maize, and oats	1750	[86]
	Unprocessed cereals	1250	
	Cereal flour for human consumption and dry pasta	750	
	Bread, pastries, breakfast cereals, and cereal snacks	500	
	Cereal-based foods and baby foods for infants and young children	200	
United States Food and Drug Administration	Flour, germ, and bran derived from wheat cereal intended for human consumption	1000	[87]
Food and Agricultural Organization of the United States/Codex Alimentarius	Raw grains such as maize, wheat, and barley	2000	[87]
	Maize, wheat, and barley-derived foods (flour, meal, semolina, and flakes)	1000	
	Cereal-based foods and baby foods for infants and young children	500	
Republic of Korea	Grain and their processed foods	1000	[92]
	Corn and their processed foods	2000	
	Cereals	500	
China	Corn, corn flour (grits, flake)	1000	[93]
	Barley, wheat, cereal, wheat flour	1000	

## 5. Occurrence of Type B Trichothecenes and DON-3G in Cereal Grains and Their Products

As previously mentioned, *Fusarium* fungi can colonize crops at the pre-harvest stages of production. Thus, it has become difficult to avoid trichothecenes contamination due to the considerable influence of abiotic conditions [11,12]. In this respect, type B trichothecenes are considered the most widespread group of *Fusarium*-generated mycotoxins, which commonly infect a wide range of cereal grains including wheat, corn, oats, barley, rice, rye, sorghum, and their products [19,94]. It is generally accepted that the geographic distribution of type B trichothecenes-producing fungi (*F. graminearum and F. culmorum*) can be influenced by climatic factors. For instance, *F. culmorum* is mostly found in cooler areas, including Western Europe, while *F. graminearum* is typically present in hot, tropical climates, including Africa, eastern Asia, eastern Australia, eastern Europe, and North America [37].

### 5.1. Wheat

Wheat crops are readily susceptible to infestation with *Fusarium culmorum* species during the heading-to-flowering phases when climatic conditions are favorable for their growth, thereby causing the destructive *Fusarium* head blight (FHB) disease in wheat [93]. Many countries monitor the presence of type B trichothecenes, particularly DON in wheat and their products, and recent occurrence studies have shown their common prevalence in these food commodities (Table 2). Liu et al. [95] surveyed the prevalence of DON, 15-ADON, and 3-ADON in 672 wheat samples from China during 2012–2013. DON was identified as the most prevalent mycotoxin (91.5%) at contamination levels ranging from 2.4 to 1130 μg/kg (mean value: 178 μg/kg). Meanwhile, the incidence rates and levels of 15-ADON and 3-ADON were considerably less than those of DON. 15-ADON and 3-ADON were detected in only 18.0% (mean value: 2.1 μg/kg, range: 0.62–6.0 μg/kg) and 1.6% (mean value: 1.3 μg/kg, range: 1.5–2.6 μg/kg), respectively. Only 0.3% of the total wheat samples contained DON at levels above the regulatory limit of China for DON in wheat (1000 μg/kg). In another recent research from China, extremely high values (up to 86,255 μg/kg) of DON were reported for wheat with a 100% frequency [96]. Bryła et al. [97] investigated the presence of DON, DON-3G, and NIV in 92 wheat samples collected from Polish markets. DON was the most dominant mycotoxin in the samples, while NIV showed the highest contamination levels. The incidence rates of DON, DON-3G, and NIV were 83%, 27%, and 70% at contamination ranges of 5.1–373 μg/kg, 15.8–138 μg/kg, and 10.5–1265 μg/kg, respectively. Moreover, the relative proportion of DON-3G compared to DON (glucosylation percentage) ranged from 4% to 37% in positive samples. In recent years, the relationship between the influence of agricultural practices (conventional and organic) and mycotoxin contamination have been discussed by several researchers [26,84]. Gab-Allah et al. [26] evaluated the contamination of type B trichothecenes and DON-3-G in 27 wheat flour samples from both organic and conventional production in Republic of Korea. The highest incidence rates were reported for DON (96%) followed by DON-3G (96%) and NIV (85%). Meanwhile, 3-ADON, 15-ADON, and FUS-X were less frequently occurring in wheat samples at incidence rates of 11%, 18.5%, and 37% of the total samples, respectively. The contamination ranges were recorded as 0.74–154 μg/kg (DON), 0.25–24.7 μg/kg (DON-3G), 0.45–126 μg/kg (NIV), 2.3–10.3 μg/kg (3-ADON), 6.0–30.6 μg/kg (15-ADON), and 0.80–4.6 μg/kg (FUS-X). According to the results, higher incidences and concentrations of all mycotoxins were found in organic wheat samples compared with conventional ones, except for 3-ADON. Palacios et al. [98] from Argentina investigated the presence and contamination levels of DON, DON-3G, and the sum of 3 and 15-ADON in 84 wheat samples during 2012-2014. All wheat samples were contaminated with DON (100%) at concentration ranges from <LOQ to 9480 μg/kg (mean value: 1762 μg/kg). DON-3G was found in 93% of the samples at concentration ranges from <LOQ to 850 μg/kg (mean value: 198 μg/kg), whereas the acetylated DON derivatives (3 and 15-ADON) occurred less frequently in the samples (49%) at noticeably lower contamination levels (<LOQ to 190 μg/kg). The glucosylation percentage across the samples varied between 6% to 22%, and the contamination with DON was found to be affected by the geographical location and year of harvest due to varying climatic conditions.

According to the available results, type B trichothecenes and DON-3G were detected more frequently in wheat and its processed products. DON was the most prevalent mycotoxin in the samples found at exceedingly high contamination levels in some cases, thus posing a potential food safety problem. Furthermore, DON derivatives and other type B trichothecenes co-occurred with DON in the same wheat samples, which can maximize the toxicological hazard due to the synergistic effects of possible combined exposure the risk of their combined exposure, and mycotoxin interactions. Therefore, special attention should be paid to the constant monitoring of these compounds in wheat and its products in order to avoid or reduce their adverse health effects, especially those arising from human exposure to DON.

### 5.2. Corn

Representative data on the occurrence of type B trichothecenes and DON-3G in corn is listed in Table 2. Iqbal et al. [99] studied the contamination of DON in 142 winter corn and 128 summer corn, as well as their products from Pakistan. DON was detected in 61.2% and 44.5% of winter corn and summer corn samples, respectively, at contamination levels of <LOQ–2967 μg/kg (winter corn) and <LOQ–2490 μg/kg (summer corn). The results also showed that the incidence rates of DON in corn and its products from the winter and summer seasons were statistically significant except for cornbread samples. Moreover, a significant number of samples showed contamination levels of DON exceeding the regulatory limits of the EU. In Croatia, a higher incidence rate (84%) and exceedingly high contamination levels (up to 17,900 μg/kg, mean value: 2150 μg/kg) of DON were documented in maize samples [100]. Berthiller et al. [101] collected 54 maize samples from Austria, Germany, and Slovakia, and found that all the samples (100%) were contaminated with DON and DON-3G at contamination ranges of 238–3680 μg/kg (average level: 753 μg/kg) and 25.0–763 μg/kg (average level: 141 μg/kg), respectively. In a more recent study from Republic of Korea, Gab-Allah et al. [26] surveyed 25 organic and conventional corn samples for all major type B trichothecenes and DON-3-G. The incidences were recorded as 96% (DON), 96% (DON-3G), 80% (NIV), 72% (3-ADON), 60% (15-ADON), and 60% (FUS-X). The maximum contamination levels of these mycotoxins were 1223 μg/kg (DON), 419 μg/kg (DON-3G), 234 μg/kg (NIV), 11.8 μg/kg (3-ADON), 298 μg/kg (15-ADON), and 28.7 μg/kg (FUS-X). According to the results, higher incidences and concentrations of these trichothecenes were found in organic corn samples compared with conventional ones, and the glucosylation percentage reached up to 35% in contaminated corn samples. In another recent research, Gab-Allah et al. [102] investigated DON, NIV, and DON-3G in corn samples collected from Egypt. Among these mycotoxins, DON was the most common (83.3%) followed by NIV (74.1%) and DON-3G (40.7%), with maximum concentration levels of 853 μg/kg, 462 μg/kg, and 257 μg/kg, respectively. In Southwest Nigeria, DON was found in maize at an incidence level of 22.2% within the range of 9.6–745 μg/kg and its modified 3-ADON was detected at an incidence level of 17.2% (0.70–72.4 μg/ kg) [103], while DON was found within a noticeably very low range of 0.10–0.70 μg/kg in maize from Northeast Nigeria [104]. EFSA also reported a high frequency of DON occurrence (70 in 136 samples, 51.5%) in maize for human consumption, with an average value of 238 μg/kg [90]. Other studies revealed that maize samples were contaminated with DON at incidence rates of 32.4% (27.0 to 2210 μg/kg) in Serbia [105], 21.4% (3.0 to 428 μg/kg) in Italy [106], 63.0% (68.0 to 2196 μg/kg) in Tanzania [107], 50.8% (<LOQ to 90.0 μg/kg) in Poland [108], 86.0% (225 to 2963 μg/kg) in Hungary [109], 24% (10.0 to 1070 μg/kg) in India [110], and 71% (215 to 278 μg/kg) in Croatia [111].

Based on the available data on corn and its products, type B trichothecenes and DON-3G were detected in varying concentrations with the highest positive rates and concentrations being registered for DON. Still, the necessity for more studies that evaluate the co-contamination of corn and its products with DON and other type B trichothecenes is of prime importance to avoiding or reducing the risk arising from the intake of these toxins from contaminated corn and its processed products.

### 5.3. Oats

Table 2 summarizes representative data on the prevalence of type B trichothecenes and DON-3G in oats. Nathanail et al. [14] investigated the contamination of trichothecenes in 31 oat samples collected from Finland. All samples were contaminated with DON at high levels up to 23,800 μg/kg (mean value: 2690 μg/kg). The incidences of DON-3G, 3-ADON, and NIV were 87.1%, 77.4%, and 71.1%, respectively, with maximum contamination levels of 6600 μg/kg (DON-3G), 2700 μg/kg (3-ADON), and 4940 μg/kg (NIV). The frequency and contamination levels of these mycotoxins in oat samples were more extreme than those in wheat and barley, with 32% of the tested oat samples containing DON levels beyond the legislative MRL for unprocessed oats (1750 μg/kg). In more recent research, Tarazona et al. [112] from Spain stated that 22.0% and 3.0% of oat samples (*n* = 100) contained detectable DON and 3-ADON, with the maximum levels of 736 μg/kg and 42.6 μg/kg, respectively. In another study conducted on oat bran from Spanish markets, a total of 17% of the samples were contaminated with DON at the maximum level of 230 μg/kg. Schöneberg et al. [113] collected 325 oat samples from Switzerland during 2013–2015 and found that 49% and 64.3% of samples were contaminated with DON and NIV, respectively, at maximum contamination levels of 1328 μg/kg (DON) and 1653 μg/kg (NIV). They found that the average contamination with DON was the highest in 2013, while NIV showed the highest levels in 2015. DON was also detected in a total of 60% (15/25) of oat samples from China with a concentration range varying between 16.8 and 244 μg/kg [114]. Islam et al. [115] from Canada analyzed 168 oat samples during 2016–2018 and documented that NIV and DON were the predominant mycotoxins in the samples with incidences of 92.0% and 55.3% and maximum concentrations of 795 μg/kg and 4143 μg/kg, respectively. Juan et al. [83] detected DON, 3-ADON, FUS-X, and NIV in 57.0%, 14.2%, 42.8%, and 57.0%, respectively, in oat samples (*n* = 7) collected from Italy at levels ranging from 10.3 to 83.0 μg/kg (DON), <LOQ to 5.2 μg/kg (3-ADON), 26.0 to 75.0 μg/kg (FUS-X), and 45.5 to 50.4 μg/kg (NIV). Previous research from Republic of Korea showed the absence of DON and DON-3G in oat samples, while NIV was detected in only 9.1% of the samples with a mean value of 23.5 μg/kg [116]. In contrast, higher incidence rates and contamination levels were recorded for DON and NIV in oat samples collected from Sweden, where DON and NIV were presented in 95.0% and 91.5% of the samples with the ranges of 99.0–5544 μg/kg and 18.0–1743 μg/kg, respectively [117]. In another research, Edward at al. [118] surveyed the prevalence of NIV, FUS-X, and DON in 303 oat samples collected during 2006–2008 in the United Kingdom. NIV was the most abundant mycotoxin with an incidence rate of 73% and a maximum concentration of 741 μg/kg. DON was found in 32% of the samples at higher contamination levels with a maximum value of 1866 μg/kg. Only 1% of the samples contained FUS-X with a maximum concentration of 18 μg/kg. In Malaysia, Soleimany et al. [119] documented that 30% of oat samples were found to be positive for DON with concentrations ranging from 22.7 to 100 µg/kg. The same incidence rate (30%) was registered for DON in oat samples collected from Slovakia, but with relatively higher contamination levels (up to 490 µg/kg) [120].

In summary, DON and NIV were the most dominant type B trichothecenes in oat samples. Meanwhile, the occurrence of other type B trichothecenes (3-ADON, 15-ADON, FUS-X) needs to be studied more thoroughly.

### 5.4. Barley

Representative studies on the occurrence of type B trichothecenes and DON-3G in barley are summarized in Table 2. Nathanail et al. [14] surveyed the occurrence of some *Fusarium* mycotoxins in 34 barley samples collected from Finland. Among all mycotoxins, DON showed the highest incidence rate in the samples at concentrations up to 802 μg/kg (mean value: 234 μg/kg). The incidences of DON-3G, NIV, and 3-ADON were 73.5%, 73.5%, and 41.2%, respectively, with maximum contamination levels of 594 μg/kg (DON-3G), 262.0 μg/kg (NIV), and 18.3 μg/kg (3-ADON). The frequency and contamination levels of these mycotoxins in barley samples were the lowest among other tested cereal grains (wheat and oat), and no barley sample exceeded the maximum regulatory levels established for DON (1250 μg/kg). However, barley revealed the maximum DON glucosylation capacity among all tested cereals. In turn, Ok et al. [121] from Republic of Korea found DON in a total of 54% (38/70) of barley samples with a concentration range varying between 3.7 and 36.8 μg/kg (mean value: 9.4 μg/kg). In another study from Republic of Korea, Ok et al. [84] surveyed 39 barley samples for type B trichothecenes. NIV was the most frequently detected mycotoxin in the samples followed by DON and 15-ADON. The incidences were recorded as 59% (NIV), 56% (DON), 15% (FUS-X), 31% (15-ADON), and 26% (3-ADON), while the maximum contamination levels of these mycotoxins were 101 μg/kg (NIV), 40.1 μg/kg (DON), 9.9 μg/kg (FUS-X), 7.1 μg/kg (15-ADON), and 3.9 μg/kg (3-ADON). A similar trend was documented by Lee et al. [116] who found NIV, DON, and DON-3G in Republic of Korea barley samples at incidences of 40.0%, 33.3%, and 13.3%, with contamination ranges of 17.3–230 μg/kg (mean value: 90.2 μg/kg), 11.7–286 μg/kg (mean value: 75.8 μg/kg), 18.0–20.6 μg/kg (mean value: 19.3 μg/kg), respectively. Juan et al. [83] evaluated the contamination of type B trichothecenes in barley samples originating from Italy. 3-ADON and 15-ADON were not detected in any sample. Whereas the frequency of DON, FUS-X, and NIV were 11.0%, 44.4%, and 33.3%, respectively, at levels ranging from <LOQ to 35.3 μg/kg (DON), 27.5 to 47.3 μg/kg (FUS-X), and 21.7 to 106 μg/kg (NIV). In another research, Soleimany et al. [7] from Malaysia found DON in 50% of barley samples with concentrations varying between 27.9 µg/kg to 72.5 µg/kg. In another recent study, Bryła et al. [122] detected DON, DON-3G, and NIV in barley-derived beer collected from Poland. Fractions of positive samples were recorded as 83%, 67%, and 56% for DON, DON-3G, and NIV with mean concentrations of 9.0 µg/L (range: 1.0–73.6 µg/L), 9.2 µg/L (range: 2.0–35.8 µg/L), and 2.4 µg/L (range: 0.50–27.6 µg/L), respectively. They suggested that the higher contamination incidence of DON-3G is attributed to the glucosyltransferase enzyme activity during the grain malting process. Additionally, Tima et al. [109] identified DON and other *Fusarium* mycotoxins in barley samples originating from Hungary. They found DON in 48% of the samples (range: 240–429 μg/kg). They also noted that DON was the most common mycotoxin in these samples followed by T-2 and zearalenone (ZEN). Mishra et al. [110] investigated 25 barley samples from India. DON was identified in 4 samples at concentrations ranging from 30.0 to 530 μg/kg (mean value 210 μg/kg). From this study, the frequency and contamination levels of DON in barley samples were less extreme than those of wheat and maize [110]. In a more recent study, DON was found in ninety-four percent (94%, *n* = 76) of Brazilian barley samples with a high contamination level over the range from 310 to 15,500 μg/kg (mean value: 5000 μg/kg) [85]. DON was also found in 53% (18/34) of Croatian barley samples at levels varying between 74.0 µg/kg and 228 µg/kg (mean value: 142 µg/kg) [111]. In Tunisia, a total of 72 commercial barley samples were collected in 2009, where fifty-seven percent of all samples were positive for DON, with contamination levels ranging from 500 to 3500 µg/kg (mean value: 1520 µg/kg).

Based on these occurrence studies, the presence and concentrations of type B trichothecenes in barley from different countries were inconsistent. Further studies are highly required to evaluate their current contamination status in various geographical regions.

### 5.5. Rice

Table 2 summarizes representative data on the prevalence of type B trichothecenes in rice. Golge et al. [123] from Turkey collected 20 paddy rice samples to explore DON contamination. DON was detected in the samples at an incidence rate of 35% (7/20) with concentrations ranging from 136 to 256 µg/kg (mean value: 195 µg/kg). Among several kinds of cereal tested in that report, paddy rice showed the highest contamination frequency for DON [123]. In recent research focused on one hundred eighty polished rice samples from Pakistan, NIV and DON were present in 28% (<LOQ to 116 μg/kg), and 8% (<LOQ to 115 μg/kg) of the samples, respectively [124]. The mean concentration levels were reported as 13.8 μg/kg for NIV and 6.9 μg/kg for DON. Ok et al. [84] evaluated the contamination of type B trichothecenes (NIV, DON, FUS-X, 15-ADON, and 3-ADON) in rice (*n* = 65), glutinous rice (*n* = 11), and brown rice (*n* = 48) collected from Republic of Korea markets. The highest incidence rates of positive samples were recorded for NIV (from 35 to 64%), followed by 15-ADON (from 25 to 56%) and DON (from 15 to 33%). Maximum contamination levels (up to 45.4 μg/kg) were also registered for NIV. In another recent study from Republic of Korea, Ok et al. [125] investigated NIV and DON in two different rice (i.e., white rice, *n* = 241 and brown rice, *n* = 241). Generally, NIV was identified as the predominant mycotoxin in the samples with higher contamination levels than DON, particularly in brown rice samples. In white rice samples, NIV and DON were detected in 21% (12.6 to 2175 μg/kg) and 5% (7.1 to 372 μg/kg) of the samples, respectively. Meanwhile in brown rice, these mycotoxins were detected in 34% and 7%, respectively, with concentrations in the ranges of 17.3–2534 μg/kg (NIV), and 9.1–435 μg/kg (DON). It should be noted that NIV co-occurred with DON in 9.1% and 14.9%, and 41.5% for white rice, brown rice, respectively. In Spain, 23 rice samples were screened for DON, 3-ADON, FUS-X, and NIV [126]. These samples were only positive for DON at a maximum contamination level of 5.5 μg/kg (mean level: 5.0 μg/kg). DON was also detected in 70.7% (30/41) of rice samples originating from Nigeria, but at very low concentrations (below 1.0 μg/kg) [104]. In another study from Nigeria, a total of twenty-one rice samples were screened for DON with only five samples found positive for this mycotoxin and the average value and the range were reported as 18.9 μg/kg and 11.2–112 μg/kg, respectively. Soleimany et al. [119] from Malaysia showed that DON was found in 26% (13/50) of rice samples with concentrations varying between 12.5 µg/kg to 81.2 µg/kg. In another report, Moreira et al. [127] identified DON and acetylated DONs in 5.3% (5/93) and 73.3% (66/93) at levels up to 125 μg/kg and 17.0 μg/kg, respectively, in rice flour samples collected from Brazil.

In summary, the most prevalent type B trichothecenes in rice from the majority of studies could be NIV followed by DON, unlike other previously mentioned cereals. While their contamination levels were moderately low, extreme concentrations were also noticed in some positive samples. Thus, special consideration should be paid to the investigation of their occurrence in this food commodity. Furthermore, studies on monitoring other type B trichothecenes (e.g., ADONs, FUS-X) and DON-3G in rice are rather scarce and further efforts are required.

### 5.6. Sorghum and Rye

Mycotoxin contamination of sorghum and rye is documented less often than contamination of other grains; however, some occurrence studies investigating certain mycotoxins in these food commodities do exist. Representative data on the prevalence of type B trichothecenes and DON-3G in sorghum and rye are listed in Table 2. More recently, Lee et al. [116] detected DON, DON-3G, and NIV in sorghum samples collected from Republic of Korea at incidence rates of 100%, 41.7%, and 91.7%, respectively. The concentrations of DON, DON-3G, and NIV were in the ranges of 18.9–712 μg/kg (mean level: 119 μg/kg), 10.4–43.4 μg/kg (mean level: 18.8 μg/kg), and 4.6–146 μg/kg (mean level: 45.3 μg/kg), respectively. In another study, the presence of DON, 15-ADON, DON-3G, NIV, and FUS-X was investigated in 110 sorghum samples originating from Nigeria [128]. The percentage of positive samples for DON, 15-ADON, and DON-3G were 3%, 2%, and 23%, respectively, and none of the samples were contaminated with NIV and FUS-X. The maximum contamination levels for DON, 15-ADON, and DON-3G were 119 μg/kg (mean level: 100 μg/kg), 44.0 μg/kg (mean level: 39.0 μg/kg), and 63.0 μg/kg (mean level: 24.0 μg/kg), respectively. In recent research from Nigeria, Olopade et al. [129] analyzed twenty sorghum samples to investigate the occurrence of DON, 3-ADON, and 15-ADON. However, none of the tested samples were found to be positive for these mycotoxins. In another study devoted to assessing the safety, occurrence, and contamination levels of mycotoxins in 1533 sorghum grain from four sub-Saharan African (SSA) countries (Sudan, Ethiopia, Mali, and Burkina Faso), only seven samples (0.46%) contained DON with a contamination range varied between 40.0 μg/kg and 112 μg/kg (mean level: 63 μg/kg) [130]. In Ethiopia, a total of 33 sorghum samples and other grains were collected and screened for DON, NIV, and other major mycotoxins. DON and NIV were identified in 90.9% (range: 50–2340 μg/kg, mean level 70.0 μg/kg), and 9.1% (range: 50–490 μg/kg, mean level: 307 μg/kg), respectively [131]. The results from that study showed high incidence and contamination levels of mycotoxins in sorghum, which might be attributed to elevated grain moisture contents due to inappropriate storage conditions. Out of 12 sorghum samples recently analyzed in Togo, 17% (2/12) were found positive for NIV and DON with concentrations in the ranges of 47.6–51.4 μg/kg (mean level: 49.4 μg/kg), and 19.0–32.8 μg/kg (mean level: 4.8 μg/kg), respectively [132]. Generally, the occurrence of mycotoxins and their contamination levels in sorghum were noticeably lower than those of maize analyzed in the same study. In another study that included four sorghum samples collected from Tunisia, DON was not detected in the samples, while NIV was found in all samples at a contamination range varying between 418 μg/kg and 667 μg/kg [133]. In another study from Tunisia concerned with the analysis of 60 sorghum samples, no type B trichothecene mycotoxins were detected in the samples, whilst the most dominant mycotoxin was enniatin B followed by ochratoxin A and enniatin B1 [134].

Juan et al. [83] evaluated the contamination of type B trichothecenes in 11 rye samples collected from Italy. They detected DON, FUS-X, and NIV in 45.4%, 45.4%, and 27.3% of the samples, respectively, at concentration levels ranging from 16.5 to 79.6 μg/kg (DON), 42.4 to 70.2 μg/kg (FUS-X), and 33.9 to 34.4 μg/kg (NIV). Martos et al. [135] investigated the occurrence of DON and its acetylated derivatives in 15 rye grain samples collected from Canada. All tested samples were positive for DON with concentrations varying between 87.0 μg/kg and 500 μg/kg (mean level 270 μg/kg). However, none of the samples contained detectable levels of the acetylated derivatives of DON. In a study preformed on 61 rye samples from Germany to investigate the presence and contamination levels of trichothecenes, all rye samples were contaminated with DON (100%) at a maximum contamination level of 288 μg/kg (mean value: 28.0 μg/kg) [136]. 3-ADON, 15-ADON, NIV, and FUS-X were identified less frequently in 59%, 80%, 3.3%, and 1.6% of the samples with maximum concentrations at 5.0 μg/kg (mean value: 0.39 μg/kg), 8.6 μg/kg (mean value: 0.73 μg/kg), 1.8 μg/kg (mean value: 0.06 μg/kg), 1.8 μg/kg (mean value: 0.01 μg/kg), respectively. In Poland, Stuper-Szablewska and Perkowski [137] conducted an analysis of type B trichothecenes in 378 grain samples including oats, rye, barley, triticale, and wheat collected during 2006–2008. The results of this study demonstrated that the highest mean concentrations of DON (46.0 μg/kg and 53.0 μg/kg) were detected in rye grain samples in 2006 and 2007, respectively. Other type B trichothecenes (FUS-X, 3-ADON, 15-ADON, and NIV) were detected at noticeably lower mean concentrations in the ranges of 1.0–27.0 μg/kg (2006), <(LOD)–26.0 μg/kg (2007), and <LOD–38.0 μg/kg (2008). Rasmussen et al. [138] from Denmark reported on the contamination of 59.4% and 13.0% rye grain samples with DON and NIV at maximum concentrations for positive samples of 257 μg/kg and 48.0 μg/kg, respectively. Furthermore, high concentration values of DON (up to 10,760 μg/kg) were reported in rye grain from the USA [139].

Based on these occurrence studies, the incidence of type B trichothecenes in sorghum and rye was inconsistent in different countries, while their concentrations seem low (below the regulatory limits of the EU), except for a few studies in which the levels of some positive samples were relatively high. Furthermore, contamination studies on type B trichothecenes in these commodities are rather scarce and further efforts are highly required to compare their recent contamination status in different areas.

## 6. Sample Preparation Methods for Analysis

**Figure 3:**
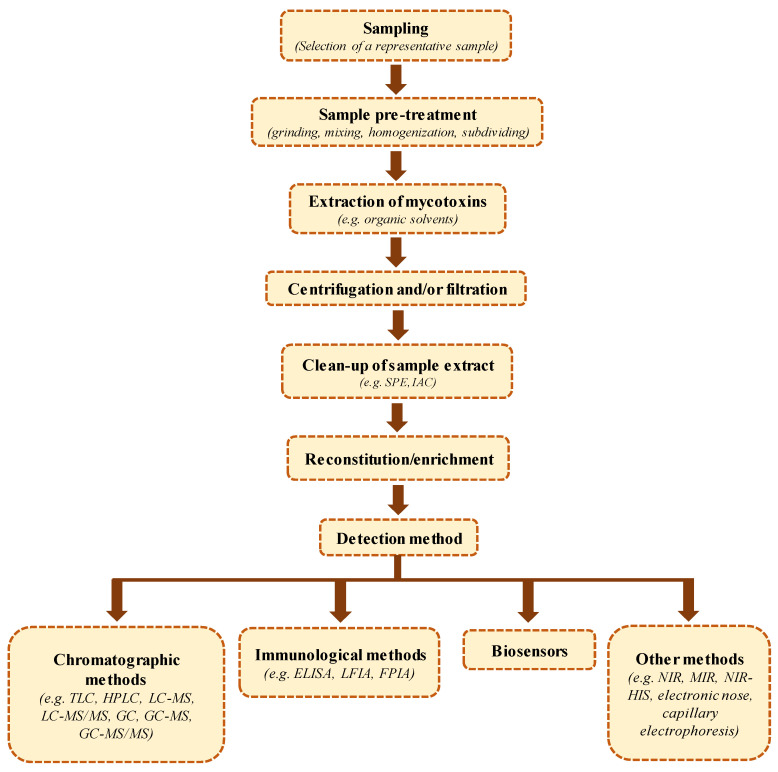
Diagram of the main steps employed in the analysis of mycotoxins in food commodities. Abbreviation: SPE: solid-phase extraction; IAC: Immunoaffinity column; TLC: thin layer chromatography; HPLC: high-performance liquid chromatography; LC-MS: liquid chromatography–mass spectrometry; LC-MS/MS: liquid chromatography–tandem mass spectrometry; GC: gas chromatography; GC-MS: gas chromatography–mass spectrometry; GC-MS/MS: gas chromatography–tandem mass spectrometry; ELISA: enzyme-linked immunosorbent assay; LFIA: lateral flow immunochromatographic assay; FPIA: fluorescence polarization immunoassay; NIR: near-infrared spectroscopy; MIR: mid-infrared spectroscopy; NIR-HIS: near-infrared spectroscopy–hyperspectral imaging.

Due to the restrictive regulations established regarding the maximum levels of trichothecenes in foodstuffs, the development of sensitive, accurate, precise, and reliable analytical methodologies is increasingly required to enforce the current regulations with a certain confidence. Sample preparation and analytical methods for the determination of trichothecenes in food and feed have been extensively reviewed [7,22,161,162]. The quantitative analysis of type B trichothecenes in food matrices generally comprises several steps: sampling, homogenization, extraction, clean-up that often includes analyte enrichment, and finally separation and detection using various instrumental and non-instrumental techniques (see Figure 3) [161,163]. Eventually, the method’s suitability for the specified analytical objective has to be assessed with appropriately adopted quality assurance procedures [164,165].

### 6.1. Sampling Tactics

Establishing an appropriate sampling procedure is crucial for analytical methodologies developed for the determination of chemical contaminants in foods due to the complexity of food matrices [166]. Collecting representative samples for mycotoxin measurements is a vital step and has a considerable effect on the final analytical results since the overall testing error includes the sum of errors across all analytical processes starting from sampling [167]. It is important that the sample used for the laboratory analysis be representative of the raw starting material, which is typically challenging for mycotoxins owing to the considerable variability of their distribution in contaminated commodities and inconsistent development of mycotoxigenic fungi on food matrices [161,166]. Therefore, a regular aspect of all sampling procedures is that the entire original sample should be pulverized and blended so that this sample and the test portion used for the analysis can contain the same concentration of the target toxins. This is particularly essential in raw grains because some toxins such as DON are mostly contained in the grain pericarp [166]. By increasing the sample size required for the analysis, the overall analytical bias might be further minimized. The homogenization degree of the sample should be also considered since it impacts the analyte extraction yield [168]. Various sampling procedures have been developed, and the common procedure commonly reported in the scientific literature has been established by the EU (Community Regulation No. 401/2006) for the official control of certain mycotoxins in foodstuffs [169].

### 6.2. Extraction Methods

The quantitative determination of type B trichothecenes in food matrices is not straightforward. Many factors must be carefully considered such as sample collection, handling, storage, and particularly the sample preparation process. Generally, the development of suitable sample preparation methodologies is often recognized as the most challenging step in the entire analytical chain because it remains the most labor-intensive and bias-prone step [170,171,172,173]. At present, a broad spectrum of extraction and clean-up procedures has been developed for type B trichothecenes to separate these mycotoxins from solid food matrices into a liquid phase and to enable their purification and enrichment before analysis. The choice and optimization of extraction solvent are made wisely by considering the physicochemical properties of mycotoxins and food sample matrices, the type of the selected clean-up procedure, and the employed separation and detection technique [7,161]. During the extraction process, the analytes will move into the extraction solvent and the compounds of interest from the sample extract are separated for detection [174]. Two extraction methods for type B trichothecenes in food are generally employed: solid–liquid extraction (SLE), and liquid–liquid extraction (LLE). The SLE method is one of the earliest sample preparation methodologies to separate analytes by partition between two involved phases: solid matrix and extractant. This method is still one of the most commonly employed approaches for mycotoxin extraction from solid materials such as grains, cereal products, and other solid foodstuffs [15,17,18,19,136,144,145,175,176]. The second extraction method relies on liquid–liquid extraction for liquid samples like juice, milk, wine, and beer to initially separate mycotoxins by transferring them from their original liquid matrix into an extraction solvent so that they can be easily analyzed using suitable techniques [122,163,177]. Type B trichothecenes are polar or relatively polar compounds with good solubility in organic solvents, such as methanol, ethyl acetate, dichloromethane, acetonitrile, chloroform, and acetone. The use of low amounts of diluted acids (formic acid, citric acid, or acetic acid) is often advantageous for the extraction process because they can break potential interactions between the mycotoxins and other matrix constituents, such as sugars or proteins [94,161,166]. Furthermore, the addition of water in small quantities can enhance the substrate wetting and further improve the extraction efficiencies by increasing penetration of the extraction solvent into the hydrophilic material [18,161,175]. Defatting step using hexane or cyclohexane is sometimes included to minimize lipophilic components in the sample extract of high lipid content [17,178,179]. In addition to the type of extraction solvent, other crucial factors such as solvent ratio, temperature, and extraction duration should be thoroughly considered to accomplish more reliable mycotoxin determination. After solvent extraction, the sample extract is usually centrifuged or filtered prior to concentration and/or purification processes.

According to the scientific literature, the most widely used extraction solvents for extracting type B trichothecenes from various food matrices are acetonitrile/water and methanol/water mixtures [7,15,19,93,125,180,181,182,183,184]. Both of these solvent mixtures are volatile and suitable for LC analysis, which is necessary when sample extract is injected directly into the analytical technique [168]. However, in multi-mycotoxin extraction from cereals and their products, more improved efficiency of acetonitrile-based solvents has been demonstrated over methanol-based solvents [94,185,186]. In previous research, the highest recoveries for thirty-four chemically varied mycotoxins (including all major type B trichothecenes) extracted from spiked wheat samples were achieved with a high proportion of acetonitrile [185]. The recoveries of the mycotoxins ranged from 87 to 111% with the acetonitrile/water mixture, except for fumonisins and patulin (17–35%), while the equivalent methanol/water mixture provided more variable and unsatisfactory recoveries, for a large number of mycotoxins, which were generally in the range of 9–204%. Indeed, acetonitrile and methanol have relatively comparable polarities [187]. However, a possible explanation for the enhanced extraction efficiency of acetonitrile might be attributed to its more appropriate selectivity towards the nonionic mycotoxins, which is mostly based upon dipole interactions rather than acid or basic functionalities [187].

Among the acetonitrile/water mixtures, acetonitrile/water (84:16; *v*/*v*) was shown to be more efficient than other solvent compositions for extracting trichothecenes of different polarity from cereal commodities because it often provides less co-extracted matrix interferences and also demonstrates acceptable recoveries [19,94,181]. Juan et al. [94] evaluated four different extraction solvent mixtures, i.e., acetonitrile/methanol (40/60, *v*/*v*), acetonitrile/methanol (60/40, *v*/*v*), acetonitrile/water (84/16, *v*/*v*), and acetonitrile/water (16/ 84, *v*/*v*) for type B trichothecenes and other *Fusarium* mycotoxins in spiked cereal grains. The obtained results revealed that the acetonitrile/water (84/16, *v*/*v*) mixture was the best extraction solvent in terms of providing high extraction recoveries as well as minimum co-extractive matrix interferences. Moreover, Zhao et al. [19] investigated two different extraction solvents, i.e., acetonitrile/water (50/50, *v*/*v*) and acetonitrile/water (84/16, *v*/*v*), for the extraction of type B trichothecenes and DON-3G from animal feed and corn. Both extraction solvents achieved satisfactory recoveries for all target mycotoxins; however, the acetonitrile/water (84/16, *v*/*v*) mixture yielded a cleaner sample extract than the acetonitrile/water (50/50, *v*/*v*) mixture. The same findings were also observed in other similar studies for the extraction of trichothecenes from grains [172,188,189]. On the other hand, type B trichothecenes are water-soluble mycotoxins, and therefore distilled or deionized water has been frequently employed as an extraction solvent for immunochemical and other methods, which also exhibits good results for DON recovery [190,191]. Pascale et al. [192] used pure water for extracting DON and NIV from wheat and achieved good recovery values in the range of 81.0–95.0%. Similarly, Trombete et al. [193] extracted DON, NIV, and DON-3G from wheat grains using pure water with extraction recoveries varying between 84.7% and 112.3%.

In addition to the classical SLE approaches, other instrumental automated solvent extraction approaches, such as accelerated solvent extraction (ASE)/pressurized liquid extraction (PLE), supercritical fluid extraction (SFE), and microwave-assisted extraction (MAE) have been developed in recent years [167,194,195,196,197]. In the conventional SLE approaches, ultrasonic energy and/or mechanical shaking are often employed for facilitating the extraction of analytes, whereas an additional form of energy input is required in these new instrumental approaches. Therefore, they often require lower consumption of harmful solvents, shorter extraction time, and provide higher extraction yield; however, these approaches continue to be cost-intensive due to expensive equipment [198,199,200].

### 6.3. Clean-Up Methods

Following sample extraction and before analysis of the desired mycotoxins, sample clean-up is a necessary step to minimize the possible co-extracted interferences (undesirable substances such as protein, pigments, sugars, lipids, or fatty acids) from food matrices, and to facilitate reliable and robust measurements of trichothecenes. Worryingly, the presence of these substances in the final sample solutions can impact the sensitivity, selectivity, precision, and accuracy of the analysis [8,18]. Various clean-up approaches have been established, such as the QuEChERS (quick, easy, cheap, effective, rugged, and safe), liquid–liquid partitioning, solid phase extraction (SPE), dispersive solid-phase extraction (DSPE), column chromatography, multifunctional clean-up columns (MFCs), immunoaffinity columns (IACs), and ion-exchange columns. However, SPE, IACs, and MFCs have been extensively used to retain type B trichothecenes from various food matrices [18,94,125,136,175,183,184,189,191,201]. IAC contains activated solid phase support covalently bound with antibodies that can selectively interact with the desired mycotoxins from sample extracts while interfering components can be eliminated by a simple washing step. Organic solvents or antibody denaturation is subsequently used to elute the mycotoxins from the IAC. Therefore, IACs rely on extremely specific antigen–antibody interactions that allow for efficient clean-up and enhanced specificity of analytes in complex food matrices [202]. However, the shortcomings of this application include the possibility of antibody cross-reactivity with other structurally related toxins, high cost, and applicability to a single mycotoxin or fewer chemically related mycotoxins [183,191]. Ok et al. [125] developed a high-performance liquid chromatography with ultraviolet detection (HPLC-UV) method for the analysis of DON and NIV in rice and bran, in which these mycotoxins were extracted with distilled water, and then the sample extract was cleaned-up using immunoaffinity columns, obtaining recoveries for DON and NIV in the ranges of 93.1–106.2% and 86.2–106.6%, respectively. In another study, Pascale et al. [192] determined the same mycotoxins in wheat by UPLC-PDA after water extraction, and they achieved recovery values from 81.0 to 88.0% for NIV and from 85.0 to 95.0% for DON. More recently, Gab-Allah et al. [18] proposed the use of immunoaffinity columns for the clean-up of grain samples that were previously extracted with deionized water. The IAC allowed the simultaneous determination of DON, DON-3G, NIV, and 3-ADON using LC-MS/MS with very acceptable recoveries in the range of 87.0–92.0%. In the same study, FUS-X and 15-ADON were not adequately retained on the IAC and therefore required a more appropriate clean-up tool. In another interesting work conducted by Zuo et al. [191], a novel IAC was synthesized based on hapten 3-O-hemisuccinyl-DON (3-HS-DON) conjugated to bovine serum albumin (BSA) by active ester method to separate and clean-up DON, 3-ADON, and 15-ADON after water extraction from cereals (i.e., maize, wheat, and oatmeal). Using this IAC, the recoveries of the target analytes were in the ranges of 67.5–93.8%, 63.8–113.2%, and 75.5–106.6%, respectively.

When sample analysis includes multiple mycotoxins, even from different families, SPEs and MFCs are more suitable and rather efficient. Currently, SPE and MFC-based approaches have gained more popularity due to their wide range of selectivity, high enrichment factor, simplicity, less solvent consumption, as well as effective elimination of matrix interferences [172]. Various adsorbent materials have been commercially available, including charcoal, silica, modified silica, Florisil (magnesium silicate), silica-based octadecyl silane (C18), polymers, ion-exchange resins, Celite, aluminum oxide, charcoal–alumina, NH_2_, and the selection of the proper sorbent is mainly governed by several parameters, including the nature of analytes, food matrix, extraction solvent and co-extractive interference components that could be existing in the sample extract [166,203,204]. C18, Celite, Florisil, aluminum oxide, and charcoal–alumina are commonly employed for the extraction of trichothecenes from various food materials [19,39,205]. Zhao et al. [19] established a simple and reliable methodology for the analysis of type B trichothecenes and DON-3G in animal feed by LC-MS/MS. The mycotoxins were extracted with acetonitrile/water (84/16, *v*/*v*) and the clean-up was based on improved dispersive solid-phase extraction with C18, Cleanert silica, graphitized carbon black (GCB), and primary secondary amine (PSA), in which the obtained recoveries ranged from 79.0% to 118.4%. Muscarella et al. [190] developed a method based on LC-FLD with online chemical post-column derivatization for the analysis of DON and NIV in cereals. Pure water was used for sample extraction and the performances of four conventional SPE cartridges with different sorbents, including Oasis HLB^TM^ cartridges, silica gel, immunoaffinity columns (DONtest WB^TM^, Vicam), and multifunctional cartridges were evaluated. Among these SPE cartridges, the polymer-based Oasis HLB^TM^ cartridges provided the best results in terms of purification efficiency and average recoveries (varying between 89% and 101%). Montes et al. [188] reported the application of MycoSep 227 column after extraction of DON, NIV, 3-ADON, 15-ADON, and FUS-X with acetonitrile:water (84:16, *v*/*v*) mixture from breakfast cereal samples. They found that this multifunctional column can provide satisfactory recoveries (69–110%) for all tested analytes. Zhang et al. [39] used MycoSep 226 for the cleanup of DON and DON-3G to study their fate during wheat milling and Chinese steamed bread processing, obtaining recoveries in the range of 70.1–109.4%. Additionally, Sasanya et al. [206] achieved recoveries of 96.4% for DON and 70.0% for DON-3G using a C18 cartridge for sample clean-up after extraction with acetonitrile–water (84:16 *v*/*v*) mixture to quantify DON and DON-3G in hard spring wheat by LC-UV.

QuEChERS (quick, easy, cheap, effective, rugged, and safe) method has been successfully used for the extraction and clean-up of different mycotoxins in various food matrices [19,207]. Indeed, QuEChERS was originally designed in 2003 for the analysis of pesticides residues in vegetables and fruits, but it rapidly gained broad acceptability in the comprehensive isolation of a broad variety of analytes including mycotoxins in different sample matrices [208,209]. This simple extraction approach includes an initial partitioning with acetonitrile in the presence of salts, such as sodium chloride and magnesium sulfate in either a 2:1 or 4:1 ratio. Following the partitioning step, the sample extract is cleaned-up using dispersive-SPE (d-SPE) [209]. Typically, sodium chloride is utilized to minimize the polar interferences in the extract, and magnesium sulfate is often used to dehydrate the organic phase. For the cleanup step (d-SPE), the most commonly used sorbents, whether applied individually or in combination are C18, PSA, and GCB. C18 is usually used to eliminate high lipid contents, whereas PSA is exploited to remove sugars, fatty acids, lipids, organic acids, as well as some pigments and GCB is particularly effective for eliminating co-extractive pigments [163,166,209]. QuEChERS is a straightforward, quick, and economical methodology that necessitates minimal amounts of solvent in comparison to other approaches. Over the last few years, this methodology has been used for the analysis of type B trichothecenes and other multiple mycotoxins in various food matrices, such as cereal grain, cereal products, plant-based beverages, spices, coffee, and livestock products (meat, milk, and eggs) [210,211,212,213,214,215,216]. For instance, Zhou et al. [216] proposed a facile and sensitive method based on modified QuEChERS that can quantify 10 mycotoxins, including DON, NIV, 3-ADON, 15-ADON, and FUS-X in wheat flour. The samples were mixed with acetonitrile:water (84/16, *v*/*v*) mixture followed by dispersive SPE clean-up with 50 mg of C18 and 50 mg of PSA. The method exhibited very acceptable performance characteristics and proved to be a rapid and robust tool for the determination of mycotoxins in wheat flour. QuEChERS method was also adopted for the determination of DON, 15-ADON, NIV, FUS-X, and ZEN in breakfast cereals and flours [214]. The sample was mixed with water and washed with n-hexane. Acetonitrile was then added, and the mixture was subjected to salting-out liquid partitioning using MgSO4, and NaCl, while the dispersive SPE clean-up was carried out with MgSO4 and C18. This method was favorable for its simplicity, selectivity, sensitivity, fast analysis, good recovery, and high robustness.

In recent times, the role of sample clean-up has changed due to the advancement in LC-MS/MS technology, and substantial purification is not usually required due to the high selectivity and sensitivity of modern analytical techniques. This has enabled the development of methodologies with reduced, or no sample purification and injection of unpurified sample extracts (i.e., dilute and shoot approaches). Using this strategy, rapid and straightforward multi-mycotoxin methods have been successfully established, also in the area of mycotoxin analysis [8,94,217].

## 7. Separation and Detection Techniques

Due to the widespread occurrence and serious toxicological effects of type B trichothecenes, analytical methods devoted to their detection in foods should be robust, rapid, accurate, and selective. This necessity has driven the scientific community to establish a variety of analytical methods for these mycotoxins, which have eased their monitoring and surveillance in many food materials. The analytical technique should be chosen based on the purpose of analysis; meanwhile, sensitive analytical methods are often necessary for low tolerance levels of mycotoxins in food commodities. Currently, quantitative and qualitative analyses of type B trichothecenes are usually performed using different chromatographic techniques, immunochemical methods, rapid methods, or other emerging detection technology. Table 3 provides a summary of the abovementioned methods.

### 7.1. Chromatographic Methods

Chromatographic techniques are the most widely employed for quantitative analysis of type B trichothecenes in cereals and other food matrices. These techniques are based on the separation of a mixture of chemical substances into their individual components by distribution between two phases: mobile phase and stationary phase [161]. Currently used chromatographic techniques are thin layer chromatography (TLC), high-performance liquid chromatography (HPLC), or ultra-performance liquid chromatography (UPLC) with smaller column packing material (particle size 1–2 µm), coupled with diode array detection (DAD), ultraviolet detection (UV), or fluorescence detection (FLD), liquid chromatography–tandem mass spectrometry (LC-MS/MS) and gas chromatography (GC) coupled with flame ionization detection (FID), electron capture detection (ECD), or mass spectrometry (MS) detection.

TLC is considered the oldest chromatographic technique for qualitative or semiquantitative analysis of mycotoxins by visual assessment or instrumental densitometry [163]. In previous reports, TLC was used for the detection of DON in cereal-based bakeries [218]; wheat, rye, barley, and maize [219]; maize [100]; and other human food commodities [220]. This method can provide easy, rapid, low-cost and simultaneous analysis of multiple mycotoxins; however, low selectivity and poor sensitivity and precision are the major drawbacks of this application [6]. Recently, high-efficiency thin-layer chromatography (HETLC) using nano silica gel TLC plates by fluorescence visualization under ultraviolet (UV) light has been successfully employed to survey DON in wheat flour with satisfactory recovery values and repeatability [221].

The recent trend in the determination of type B trichothecenes in food relies on the application of reliable, rapid, and simple technologies for multi-mycotoxin analysis with lower detection limits, and enhanced selectivity. As one of the most widely used separation techniques, HPLC has found widespread uses in a variety of sectors, such as scientific research, clinical analysis, environmental analysis, food analysis, and diagnostics. Coupled with UV detection, DAD, or FLD, this technique can be widely employed for the confirmatory analysis of various types of mycotoxins, including type B trichothecenes in foods. Such methods offer good sensitivity, selectivity, and precision, and are hence occasionally used as official reference methods for the validation and verification of immunochemical tests [43]. Since type B trichothecenes can absorb UV light of a certain wavelength, various studies proposed HPLC with DAD or UV for the quantification of these mycotoxins, particularly DON, NIV, and DON-3G. In recent times, HPLC-DAD and HPLC-UV have been applied for analyzing DON, DON-3G, and NIV in wheat grains [193]; DON, DON-3G, and NIV in rice and baby formula [222]; DON and NIV in rice and bran [125], DON in wheat flour, instant noodle, and biscuits [223]; DON, NIV, and DON-3G in wheat and maize [102]; and DON, NIV, together with their glucosides in cereals and related products [116]. On the other hand, HPLC-FLD in combination with appropriate extraction and purification procedures can exhibit comparable sensitivity to that attained by LC-MS/MS, as well as very good selectivity [163,224]. However, due to the lack of natural fluorescence in trichothecenes, this method necessitates a derivatization step to improve the analyte detection signal. Furthermore, detection by this technique is only applicable to a single toxin or a group of chemically related toxins [190,224]. It is worth mentioning that the HPLC-FLD method has been recommended by the European Committee of Standardization (CEN) and AOAC International for the analysis of mycotoxins in grain cereals [225]. Recently, the HPLC-FLD method has been used to determine type B trichothecenes in different agriculture commodities, including DON and NIV in wheat [226]; DON and NIV in various cereals [190]; DON and its major conjugates (DON-3G, 3-ADON, 15-ADON) in maize, wheat, and barley [224]; and DON in cereals intended for human consumption [227].

To fulfill the increasing demands of testing laboratories and to facilitate compliance with the current regulations, rapid and high throughput multi-mycotoxin analytical methods are highly required. In recent times, LC-MS/MS analysis has played an increasingly significant role in the analysis of chemically diverse mycotoxins in complex food matrices [2,10,166,228]. The most commonly used analyzer in the mycotoxin field is triple quadrupole (QqQ), which enable tandem mass spectrometry in the so-called multiple reaction monitoring (MRM) modes [166]. High chromatographic separation capacity, outstanding detection sensitivity, fast acquisition features, compatibility with a broad range of sample preparation procedures, and wide linear dynamic range are the main features of LC-MS/MS [166,172]. As a consequence, LC-MS/MS method has become the main choice for regulatory testing agencies in various countries, and the majority of research studies evolved in recent years have used this method for quantifying type B trichothecenes in various foods: DON and DON-3G in maize, groundnuts, and their products [151]; DON, NIV, DON-3G, 3-ADON, 15-ADON, and FUS-X in corn and wheat [18]; DON, NIV, 3-ADON, and 15-ADON in cereals, infant formula, spices, nuts, oil, and cocoa [229]; DON and DON-3G in wheat and maize [101]; DON and acetylated derivatives in DON wheat and maize [144]; DON, DON-3G, and NIV in animal feed and maize [148]; DON, NIV, 3-ADON, 15-ADON, and FUS-X in wheat grains [142]; DON, DON-3G, 3-ADON, NIV, and FUS-X in wheat, oat, barley, and rye triticale [24]. On the other side, the introduction of high-resolution mass spectrometry (HRMS) allows for the analysis of numerous contaminants with a single extraction, including pesticides, mycotoxins, veterinary drugs, and ergot alkaloids [167,230]. Due to its outstanding sensitivity, high resolving power, and accurate mass measurement, LC coupled with HRMS stands as a suitable method for the detection of non-targeted mycotoxins or new members of known mycotoxin groups, which is particularly useful for the discovery of metabolites or other masked forms whenever an analytical standard is not available [230,231,232]. In an interesting work, UPLC coupled with quadrupole Orbitrap HRMS was applied for the first time for the identification and detection of fusarenon X-glucoside in wheat grain artificially infected with *Fusarium* species [233]. This method was also proposed to investigate the occurrence of 54 mycotoxins in ready-to-eat tree nut products [234], and to analyze various *Fusarium* mycotoxins, including DON, NIV, DON-3G, 3-ADON, and 15-ADON in cereals (wheat, maize, and barley) [235].

The gas chromatographic (GC) method is a useful tool for evaluating volatile substances that can be vaporized without decomposition. In the past, the use of GC-FID or GC-ECD for the identification and determination of type B trichothecenes was a common practice; however, GC has been recently considered a rarely utilized technique because of its narrow scope of analysis. Eke et al. [236], and Schothorst et al. [237] proposed GC-FID methods for the simultaneous determination of type A and B trichothecenes in food and feed (wheat, semolina, and corn grits feed). Furlong et al. [238] applied a GC-FID method for the confirmation and quantification of DON, NIV, and other mycotoxins belonging to type A trichothecenes in wheat. In other studies, GC-ECD was used for the determination of DON and DON-3G in wheat [239]; DON, NIV, 3-ADON, 15-ADON and FUS-X in wheat [195]; and DON and NIV in polished rice, corn, and wheat [240]. To enhance the volatility of type B trichothecenes and enable more sensitive detection, GC techniques often require derivatizing the trichothecenes’ hydroxyl groups via trimethylsilylation or fluoroacylation processes. When GC is coupled to an MS detector (GC-MS) or tandem MS detector (GC-MS/MS), this method can serve as a very valuable analytical tool for the determination of various toxins in food with maintaining high levels of selectivity and sensitivity. In this regard, GC-MS/MS has been successfully employed in the analysis of type B trichothecenes, including DON, NIV, 3-ADON, and FUS-X in wheat semolina [241], cereal-based products (wheat, rice and maize) [126], and more recently in breadsticks [242].

### 7.2. Immunochemical Techniques

Although the abovementioned physicochemical detection approaches for type B trichothecenes benefit from high sensitivity and selectivity, lengthy sample preparation, high-priced instruments, as well as the necessity for highly qualified technicians are their major shortages [10,166]. Therefore, different immunochemical techniques have been developed as rapid and cost-effective alternatives to overcome the above limitations.

#### 7.2.1. Enzyme-Linked Immunosorbent Assay

Enzyme-linked immunosorbent assay (ELISA) approach for detecting trichothecenes is typically based on a highly specific molecular interaction between the required target and the biorecognition element (anti-trichothecene antibody or an enzyme-labeled trichothecene antibody) [166,243]. Thereafter, the resulting complex can react with a chromogenic substrate to produce a quantifiable result. Recently, ELISA methods have gained more popularity in identifying and measuring various mycotoxins in food due to their easy manipulation, fast detection, and relatively low cost; however, their inefficiency in detection at low concentrations has limited their application in certain situations [88,163]. Moreover, ELIZA kits are designed for one-time use only, which would increase the cost of detecting multiple mycotoxins in contaminated food samples [243]. Pleadin et al. [100] proposed an ELISA method for the determination of DON and ZEN in contaminated maize using a particular and sensitive anti-DON/ZEA monoclonal antibody. Regarding the DON’s sensitivity, a detection limit of 10 µg/kg was achieved, and the average recovery was as high as 92%. Moreover, a good correlation (*r* = 0.96) between DON concentrations determined by ELISA and HPLC-UV was obtained. Santos et al. [244] reported the development of an indirect competitive ELISA (ic-ELISA) using an anti-DON.3 monoclonal antibody (mAb) to investigate the occurrence of DON in wheat samples. The detection limit of the developed method was 177.1 µg/kg, and the average recovery of DON was 108.4%. Other recent studies have also reported on the use of ELIZA methods for detecting type B trichothecenes in different foods, including NIV and 15-acetylnivalenol in wheat and other grains [245]; DON in cereals and derived products (wheat, barley, and malt) [246]; DON in maize, barley, rice, wheat, oat, flour, and milk [247]; NIV and DON in wheat kernels [248]; and DON and DON-3G in cereal-based beer [25]. Nevertheless, more efforts are required to improve their sensitivity. It is noteworthy to mention that the detection accuracy with the ELISA method may be influenced by potential matrix interferences and cross-reactivity with structurally related mycotoxins or other specific matrix components co-present in sample extracts [25,41,247]. For instance, the developed antibodies for DON or DON-3G showed high cross-reactivity with acetyl derivatives of DON since they may share similar immunodominance [246,247]. Consequently, confirmatory methods based on HPLC are often needed to meet regulatory standards.

#### 7.2.2. Lateral Flow Immunochromatographic Assay

Lateral flow immunochromatographic assay (LFIA) is a quick, unique and straightforward (single-step) test format that requires no special equipment, additional chemicals, or laborious preparation processes [249,250]. LFIA depends on a competitive or “sandwiched” immunoassay using a labeled antibody as a signal reagent. It consists of three parts: a conjugate pad containing antibodies, a porous membrane (chromatographic material) and an absorbent pad [251]. After applying the liquid components of the test onto the conjugate pad, it moves across the membrane by capillary force to the absorbent pad, which absorbs the excess reagents and prevents the backflow of the liquid. Antibody-labeling tag conjugates, such as quantum dots, gold nanoparticles, and luminescent nanoparticles are currently used as the labeled reagents that could interact with the pathogens in the moving liquid sample. Visual detection results can be obtained in a short time through direct color markers or enzymatic color reactions, which makes this application an excellent onsite detection technology [252].

Although LFIA has been mainly employed for qualitative detection, the emergence of modern technologies allows for the generation of quantitative measurements. Due to the aforementioned features, the quantitative LFIA is particularly attractive in the food safety area for deoxynivalenol measurements, particularly for onsite testing and first-level screening. Liu et al. [249] established an LFIA test using DON-V lateral flow strips (consisting of a reader, barcode scanner, and strip cassette) for the detection of DON in durum wheat, semolina, and pasta. Limit of detection (LOD) was recorded as 0.30 mg/kg, and the results obtained from this simple approach were comparable to those of LC-MS measurements. Overall, the developed LFIA test proved to be a rapid, easy, and inexpensive onsite screening method for the quantitative detection of DON in wheat. In a more recent study, Yu et al. [250] reported the development of an enhanced LFIA test using silver staining as a signal amplification strategy to simultaneously detect DON and fumonisin B1 in maize. The test was characterized by exploiting gold nanoparticle (AuNPs)-labeled antibodies to serve as a detection probe, as well as a catalyst. The method was validated by the analysis of naturally contaminated maize samples, obtaining a detection limit of 40 ng/mL for DON, and the results were in good agreement with HPLC-MS/MS measurement results. The major attributes of this system were analytical rapidity, simplicity, low detection cost, and suitability for onsite detection. Recently, the LFIA assay has been widely developed for rapid and simultaneous detection of multiple analytes. In a pioneering work, Song et al. [253] proposed a novel and rapid LFIA test using three class-specific monoclonal antibodies for the qualitative and/or semiquantitative determination of DON, ZEN, aflatoxin B1 (AFB1), and their main metabolites (DONs, ZENs, AFs) in maize and wheat. No cross-reactivity to other mycotoxin groups was observed, and the recoveries of all analytes from spiked maize and wheat varied between 80% and 120%. Furthermore, the detection limit for DON was 3 µg/kg. Other previous studies reported the application of LFIA tests for the detection of DON in different food materials, including barley [254]; rice and corn [255]; wheat, maize, and bran [256].

#### 7.2.3. Fluorescence Polarization Immunoassay

Fluorescence polarization immunoassay (FPIA), unlike the previously mentioned assays, is a homogeneous method performed in the solution phase without the need for attaching immunoreagents to solid surfaces [251]. Therefore, this method provides faster detection with no additional separation and washing steps, making it convenient for monitoring large-scale samples. In this technique, the analyte and fluorescent-labeled analyte (tracer) compete for specific antibody-binding sites in the solution. The detection is based on the fluorescence polarization of the fluorophore tracers, which is inversely proportional to their molecular rotation. In the presence of free toxins in the sample solution, the toxins bind with the available antibodies, while free tracers exist in the solution. Thus, yielding a high rate of rotation and consequently a lower fluorescence polarization value and vice versa [251,257]. Various fluorophore tracers and mAbs have been investigated for the development of FPIA tests for trichothecene detection. Maragos et al. [258] developed an FPIA technique for the determination of DON-contaminated wheat using a DON-specific monoclonal antibody and a fluorescently tagged DON (DON-fluorescein, DON-FL). The assays were very rapid and user-friendly, requiring only mixing the aqueous extract of wheat samples with the antibody and DON-FL. The authors noted that the sensitivity of the assay decreased with increasing the incubation time of the sample and the tracer. The IC_50_ for the assay reached 30 ng/mL with a tracer incubation of 15 s, while IC_50_ higher than 1000 ng/mL was recorded at an incubation time of 10 min. The results from this assay were compared favorably to an HPLC reference method; however, high cross-reactivity of the DON monoclonal antibody to 15-ADON was observed in the assay. Currently, multi-analyte FPIAs have gained growing attention because of their high throughput detection, inexpensive costs per assay, and minimal sample consumption. Li et al. [257] have recently established a novel homologous multi-wavelength fluorescence polarization immunoassay (MWFPIA) for the rapid, simple, and high-throughput detection of DON, T2 toxin, and FB1 in maize. Three different dye-labeled tracers and specific monoclonal antibodies were employed to perform the MWFPIA. Under optimal conditions, recoveries of DON from spiked maize were in the range of 78.7–103.1%, and the LOD was 242 μg/kg. Upon comparison of the measurement results of naturally contaminated maize samples obtained using the developed MWFPIA with those from the HPLC-MS/MS technique, good agreement between the two techniques was obtained with a correlation coefficient of 0.97 for DON. According to the obtained results, this MWFPIA technique proved to be a versatile strategy for food safety analysis.

### 7.3. Biosensors

Biosensors are measuring devices consisting of receptors (specific bio-recognition elements) connected to a physicochemical element (transducer), that transforms the signal generated by the bio-recognition element into a detectable response (thermal, optical, electrochemical, or piezoelectric signal) [243,259]. Antibodies, antigens, nucleic acids, proteins, cells, and enzymes have been frequently exploited as bio-recognition elements. Utilizing adsorption, trapping, or covalent bonding, these bioreceptors are successfully immobilized in the biosensor. When the toxin interacts with bioreceptors, a physicochemical signal is produced from the biochemical reaction, which can be amplified and processed by a detector before displaying it on an electronic display system [251]. This is an area of growing interest in recent years for the detection of deoxynivalenol and other trichothecenes in foodstuffs. High transmission, good selectivity, reproducibility, sensitivity, speed of analysis, and low-cost operation are the main characteristics of biosensors [260,261]. For detecting trichothecenes in food matrices, electrochemical aptasensors are often utilized, which typically comprised toxin-specific aptamers (peptide, antibody, oligonucleotide, or peptide nucleic acid) and highly sensitive transducer elements. Ong et al. [261] established a high-performance biosensing system (DON-aptasensor) using DON-aptamer (a tailor-made aptamer sequence) as the biorecognition element, and iron nanoflorets on 3D graphene–nickel substrate (INFGN) of large surface area as the transducer element. This developed system allowed for simple, selective, sensitive, and cost-effective detection of DON in food and feed with a minimum LOD of 2.11 pg/mL. Wen et al. [262] developed a facile and novel electrochemical aptasensor using a multifunctional N-doped Cu-metallic organic framework (N–Cu–MOF) nanomaterial and DON-specific aptamer for the detection of DON in wheat samples. The great electrical conductivity and the large specific surface area of N–Cu–MOF significantly enhanced stability, selectivity, and sensitivity of the electrochemical biosensing probe. The established electrochemical aptasensor device exhibited a wide linear concentration range (0.2–20 ng/mL), very low LOD (0.008 ng/mL), and good reproducibility for DON, being therefore efficient and reliable in rapid and sensitive analysis of this mycotoxin in various food materials. Moreover, the authors demonstrated that this proposed approach can be readily extended to other toxins by changing the target recognition aptamers. In recent times, aptamers have been folded into three-dimensional (3D) structures such as 3D DNA Walker, which often demonstrate smart dynamic interaction, multiple binding signal events, and outstanding predictability. For potential signal amplification, enzyme amplification strategies are often introduced into aptasensor since they are highly efficient and easy to use. Wang et al. [260] reported the development of an electrochemical aptasensing platform based on Exonuclease III (Exo III)-assisted triple-amplified for measuring DON in maize using PtPd nanoparticles polyethyleneimine-functionalized reduced graphene oxide (PtPd NPs/PEIrGO) as the transducer element, which enhanced the surface area and conductivity of the electrode. Satisfactory results in terms of stability and reproducibility were obtained, and the developed technique was applied to the detection of DON in real maize samples with an LOD of 6.9 × 10^−9^ mg mL^−1^. On the same track, a DNAzyme-assisted triple-amplified electrochemical aptasensor has recently been reported for ultra-sensitive detection of trichothecenes, through the signal amplification of nanomaterials and the interaction of Ag^+^ and DNA. The incorporation of metal ions as cofactors of high selectivity can enhance aptamer availability by cleaving nucleic acid substrate of DNA molecules [243]. Compared with protein enzymes, DNAzyme offers the benefits of economic synthesis, high stability, and easy modification [263].

Another distinctive feature of biosensors over the one-time-use ELISA kits and other fast screening strip techniques is their ability to be recycled. Although various biosensing systems have gained extensive application as efficient tools in mycotoxin analysis, the majority of these methods still require extensive sample preparation procedures and are incapable of performing simultaneous quantification of multiple analytes.

### 7.4. Infrared Spectroscopy

Infrared spectroscopy (IR spectroscopy) is an optical technique that measures the absorption of infrared radiation (800–25,000 nm) by chemical bonds in a given molecule [243]. Different chemical functional groups tend to absorb IR radiation at different frequencies. Therefore, the light transmitted through the molecule can be used for chemical structure identification, chemical fingerprinting, and quantitative measurements of chemical species. Combined with appropriate mathematical tools such as principal component analysis (PCA), the IR spectroscopy technique can provide a promising tool for screening and quantification of mycotoxins in food industries due to its attractive features, such as analytical rapidity, easy operation, and non-destructive testing with minimal or no sample manipulation [264,265]. For detecting type B trichothecenes in foods, different IR spectroscopic techniques have been successfully utilized, such as near-infrared spectroscopy (NIR), mid-infrared spectroscopy (MIR), and hyperspectral imaging [266,267,268]. In a recent study, the application of Fourier transform NIR (FTNIR) and MIR spectroscopy was investigated for the rapid analysis of DON in wheat bran samples aiming at grouping them into two classes based on the maximum regulatory limit of 750 µg/kg established for DON in cereal products by the EU [264]. Based on this classification model, overall discrimination rates were in the ranges of 87–91% and 86–87% for the FTNIR and FTMIR spectroscopy, respectively. Therefore, the results suggested that FTNIR spectroscopy coupled with an appropriate classification model is a promising and efficient approach for the quick classification of several bran wheat samples according to DON contamination. In another study, Tyska et al. [268] investigated the applicability and effectiveness of NIR (dispersive NIR and FTNIR) to analyze DON in wheat flour samples (*n* = 267) using various chemometrics techniques such as discriminatory methods, and to classify the samples into two classes according to the maximum regulatory limit of 750 µg/kg established for DON in cereal products in Brazil. The results showed overall discrimination rates in the range of 85.0–87.5% with a 10–15% error, thereby indicating that NIR can be an outstanding alternative strategy for the classification of wheat flour samples based on the levels of DON in the tested samples.

It is well-established that conventional IR spectroscopic techniques enable only a mean spectrum (average measurement) of a sample without offering information about the spatial distribution of chemical constituents across the sample since they are considered point-based scanning methods [266,267]. To address this drawback, NIR hyperspectral imaging (NIR-HIS), which integrates conventional digital imaging and NIR spectroscopy into a unique system has recently been introduced, presenting improvements on conventional NIR devices. This advanced and high-performance analytical technology provides images in a 3D form called “hypercube”, which simultaneously exhibits spatial (localization) and spectral (identification) information for each pixel in the image [266,267]. In recent years, NIR-HIS has gained wide application in the assessment of various contaminants in different food materials, as well as evaluating their quality and safety based on classification and defects detection strategies. Shen et al. [266] studied the feasibility of NIR-HSI coupled with chemometrics, and partial least squares discriminant analysis (PLS-DA) for analyzing DON in 120 wheat kernels (severely damaged, moderately damaged, and asymptomatic wheat kernels). The results revealed that this technique can provide rapid, high-throughput, and nondestructive analysis of DON. Moreover, it can identify the distribution of DON content in wheat kernels and classify these samples into different grades based on the degree of *Fusarium* infection. Another similar study based on NIR-HSI and chemometrics was developed to investigate DON presence and *Fusarium* damage in wheat kernels. The study was also devoted to the classification of the grains into two groups based on the EU maximum limit (1250 µg/kg) [267]. Based on the obtained results, the technique showed a substantial contribution to the management of *Fusarium* and DON in single wheat kernels, and also overcame their contamination heterogeneity since the classification accuracies according to symptomatology and DON levels were 100% and 98.9%, respectively.

### 7.5. Other Emerging Detection Technologies

#### 7.5.1. Electronic Nose

A further example of a rapid detection method for mycotoxins is based on the electronic sense of smell. Numerous volatile compounds are found in food odors and aromas, which can be employed as sensory markers of food quality [269]. An electronic nose (EN) is a variant of gas chromatography that attempts to mimic the human olfactory sensory system, which can exhibit rapid, non-destructive, and cost-effective detection of mycotoxins in various food matrices [163]. This approach is based on the interaction of a volatile mycotoxin with chemical sensors with different specificities, leading to the generation of a signal, which is then utilized effectively as a fingerprint of the volatile mycotoxin rising from the tested samples, which serves its identification by means of pattern recognition system [270]. Current EN applications for mycotoxins have focused on the identification of toxigenic fungal species, instead of detecting the mycotoxin itself. The ability to distinguish between toxic and nontoxic fungi is practically beneficial using this approach. A few reports have been published on the application of EN in the detection of DON in grains; wheat bran [270] and durum wheat [269,271]. Theses EN methods have been mainly based on metal oxide semiconductors (MOS) sensors and proved to be suitable for rapid, inexpensive, and user-friendly screening of large numbers of wheat samples to investigate DON contamination and distinguish the microbiological quality of these samples.

Using this technology for mycotoxin detection in food samples is still in the early development phase. Techniques need to be further improved or optimized to enhance their selectivity, reduce interferences, and enable mycotoxin detection in food samples at low concentration levels. Furthermore, many mycotoxins are nonvolatile organic substances that raise major difficulties for EN-based detection.

#### 7.5.2. Capillary Electrophoresis

Capillary electrophoresis (CE) is a chromatographic technique in which the electrochemical potential of mycotoxins is used as the basis of their separation, and UV absorbance or laser-induced fluorescence as detection systems [2,163]. The separation is achieved by the migration of charged particles in the run buffer, where cations migrate to the cathode and anions migrate to the anode under the influence of an electroosmotic flow. Different features such as reduced consumption of hazardous solvents and buffers, the use of less expensive capillaries, and reduced analysis time make this technique a feasible alternative to those necessitating HPLC [272]. Unfavorably, this approach may not be very sensitive since only small sample volumes can be tested; however, capillary zone electrophoresis in combination with laser-based fluorescence detection and appropriate purification procedures, such as IAC, can exhibit comparable accuracy, sensitivity, and precision to that acquired by HPLC for the analysis of trichothecenes in grains [273].

## 8. Advances in Management and Control Strategies

In fact, the infestation of agricultural commodities with type B trichothecenes poses a significant threat to human and animal health, as well as financial losses to the agro-food systems worldwide. These mycotoxins can be produced by mycotoxigenic *Fusarium* species that contaminate agricultural crops at the pre-harvest stages of production; hence, it might be difficult to avoid their formation due to the considerable influence of abiotic conditions [18]. Generally, mycotoxins can contaminate crops along the entire food management chain from pre-harvest to post-harvest phases and the existence of fungi does not necessarily imply mycotoxin infection, since mycotoxins are produced under conditions that are specific and distinct from fungal development [276]. In light of their deleterious impacts, the effective control of these compounds at least to the levels established by regulations has become increasingly essential. To date, various control measures and decontamination techniques have been established to minimize the growth of fungi in field crops and to detoxify mycotoxin-contaminated foods. These typically include management policies based on good hygiene practices (GHPs), good agricultural practices (GAPs), good management practices (GMPs), good storage practices (GSPs), bio-safe post-harvest decontamination methods, and appropriate quality assurance programs [274].

### 8.1. Pre-Harvest Practices

Pre-harvest precautions, for reducing *Fusarium* spp. growth and mycotoxin contamination in all phases of crop production, require the integration of different GAP measures, such as wise use of fungicides/insecticides, appropriate crop rotation, selection of plant cultivars that are resistant to insect damage or *Fusarium* spp. infection, proper irrigation and fertilization practices for nutrient enrichment, soil tillage, planting pathogen-free seeds, planting and weed eradication, planting date and harvest times, development of forecasting models, insect management, and production of genetically engineered plants for mycotoxin inhibition.

The use of agricultural chemicals (i.e., fungicides) such as triazoles, strobilurins, and carbendazim is beneficial for the management and control of *Fusarium* head blight (FHB) disease. Because different *Fusarium* species may cause FHB, the sensitivity to fungicide is often an important element in identifying the efficacy of a fungicide group [277]. Recent research has shown that plant pathogenic fungi may adapt to fungicides due to mutation, which results in their resistance against fungicides and decreased efficiency in some instances [278]. For a long time, farmers have relied on fungicides in conventional agriculture to protect their crops; however, other health hazards linked with the prevalence of fungicides in foods should be addressed via risk management protocols [279].

Crop rotation (growing alternative crops successively) can have a significant effect on enhancing soil health and reducing pathogen levels by breaking the production chain of infectious material due to the unavailability of the host plants [243]. In fact, the majority of pathogens that are present during cropping season gradually die between the interval of two or three years [243]. Therefore, crop rotation with plants that are not hosts to *Fusarium* species such as clover, alfalfa, potatoes, and other types of vegetables should be used to reduce the inoculum in the field. When wheat was planted after crops other than maize, the concentration of DON decreased by 31% when compared to maize as the pre-crop [280]. In another study, it was observed that the degree of FHB infection and DON contamination in grains varied greatly depending on whether the previous crop was maize, wheat, or soya bean [281]. The minimum concentration of DON in grains was detected after soya bean and the maximum concentration of this mycotoxin was observed after maize due to the carry-over of pathogens onto grains. In Poland, wheat planted after corn or wheat showed a greater frequency of FHB infection compared to sugar beet in pre-crop [282]. Management of planting date is another critical pre-harvesting practice for controlling mycotoxin contamination because it often determines the date of flowering and weather conditions at the flowering stage; if the date of planting coincidences with spore dispersal, the possibility of fungal attack will be more serious [278]. On the other hand, planting maize, wheat, and other grains should be also planned to prevent increased rainfall and excessive moisture during the flowering stages since these conditions often have a favorable effect on FHB infection and type B trichothecenes production [283].

Excessive heat and soil dryness during the seed’s growth and maturity can induce plant stress, which adversely affects the resistance of grain to fungal infection and mycotoxins contamination [284]. The application of fertilizers, especially those that are rich in nitrogen was found to promote the development of *Fusarium* species while also promoting plant growth, which in turn leads to an increase in trichothecene contamination [285]. For instance, maize planted in organic farming has a 50% lower *Fusarium* infection rate than that grown in conventional farming [286]. In former research, Lemmens and coworkers in Austria found a substantial rise in FHB infection and DON contamination in grains by increasing a mineral N fertilizer from 0 to 80 kg/ha [287].

Recent studies have shown that land preparation strategies such as crop rotation and soil tillage are among the most essential pre-harvest practices that can influence/control DON production in wheat and maize [283]. Soil tillage with exposure to autumn sunshine was revealed to be a promising strategy that can assist in destroying *Fusarium* inoculum and decreasing the production of trichothecene mycotoxins due to the destruction of infected crop residues that can transfer the infection to the subsequent crop [288]. The selection of plant cultivars is very critical in disease management and should be performed based on their resistance to mold infestations. Recent investigations have shown that planting pathogen-free seeds or selecting genetically modified plant cultivars that are resistant to *Fusarium* spp. infection can lessen the invasion of mycotoxigenic fungi in crops [289]. In addition, some recent and pioneering techniques such as gene silencing through RNA interference (RNAi) have been employed as an efficient strategy based on genetic engineering to mitigate DON production by *F. graminearum*, and this strategy can be used for the mitigation of other mycotoxins as well [290]. However, the introduction of foods derived from genetically modified crops should be accompanied by adequate policies to guarantee consumer safety and these foods are still not well-accepted by many countries around the world [291].

### 8.2. Harvest and Post-Harvest Practices

The selection of an appropriate time for grain harvesting is important to ensure the production of healthy grains with a good degree of maturity to maintain the integrity of the seeds since the presence of a large number of immature and overmature seeds can enhance mycotoxin contamination in the final products [292]. In rainy seasons, harvesting should be accomplished in the shortest time possible to reduce fungal development. During harvesting, crop stress should also be reduced by avoiding earlier harvesting, mechanical damage, collection of the damaged kernel, and contact of kernels with soil [293].

Post-harvest management strategies are important in preventing mycotoxin contamination in food raw materials, particularly control of storage and distribution conditions, sorting (i.e., elimination of damaged or infected crops), and application of various decontamination strategies [278]. During transportation, the transport vehicle and containers should be dry, clean, and free from fungi, insects, or any other contaminants. After grain harvesting, moisture content and temperature play a significant role in determining the occurrence of mycotoxigenic fungi, the level of colonization, and the subsequent accumulation of relevant mycotoxins in the grains [29,30,31]. Therefore, appropriate grain drying and proper storage under controlled conditions of temperature, moisture, relative humidity of the grains (<15%), as well as pest or insect management are among the most important measures that control/mitigate fungal growth and mycotoxin formation in foods [88]. Drying groundnuts up to 6.6% moisture content ensured the nuts were free of fungi species and mycotoxins for six months while reducing the moisture content of corn to 15.5% lowered the hazard of fungal growth and mycotoxin formation [294]. The drying process should be performed using solar dryers rather than sun drying because slow drying enhances mycotoxin contamination [295]. It has been demonstrated that high storage temperatures and water activity can promote the growth of *F. boothii*, *F. graminearum sensu stricto*, and *F. meridionale*, and further generation of DON and NIV [296]. The effect of water activity and temperature on the growth of various *Fusarium* species, including *F. culmorum*, *F. avenaceum*, *F. tricinctum*, and *F. poae* was also studied with temperatures of 5–10 °C, and water activity of 0.90–0.91 a_w_ yielding the lowest mold growth [297].

Various decontamination techniques have been explored to minimize or remove type B trichothecenes in raw food materials and are mainly categorized into physical, chemical, and biological methods [182].

**Physical Methods:** the most commonly used physical methods to detoxify trichothecenes include washing, cleaning, density segregation, sorting, milling, dehulling, sieving, thermal inactivation, ultrasound treatment, radiation treatment, bonding with the bacterial cell walls, and adsorption [182,243]. However, the concentration and dispersion of mycotoxins throughout the seed can affect the effectiveness of these approaches. *Fusarium* infection often initiates from the external layer of the grain; hence, the mycotoxins are mostly found at the surface of the grain. Therefore, milling procedures can be efficient for the removal of mycotoxins from grains [175]. Vidal et al. [40] observed a reduction of DON content in the final flour or semolina after milling wheat grains, while an increase in DON content was detected in the external parts of the grains (shorts and bran).

Thermal treatment in normal food processing operations can be performed either by dry processes (roasting, baking, and frying) or wet processes (cooking and steaming). The degree of mycotoxin degradation by heat treatments is highly influenced by multiple factors such as temperature, moisture content, and heating duration. Trichothecenes are normally stable at 120 °C; however, they will partly decompose when the temperature rises over 200 °C. Vidal et al. [298] detected a considerable decrease in DON content due to the thermal process of baking. DON reduction was more pronounced at greater initial DON concentrations and higher temperatures. They also found an increase in DON-3G content (>300%) under mild baking conditions, while it was rapidly reduced under harsh conditions. In another study, Zhang et al. [42] reported on the reduction of DON and DON-3G levels in noodles after cooking due to the leaching of theses mycotoxins into the cooking water. Generally, this process is regarded as an important strategy for the mitigation of type B trichothecenes in food materials; however, the residual degradation products can have harmful effects that are different from those induced by the original toxin [299]. On the other hand, UV irradiation has been commonly utilized as an efficient approach to remove mycotoxins because of its reduced secondary contamination and lesser influence on degradation production. On the same track, gamma radiation (γ) has also been employed as a viable tool for the preservation and maintenance of food quality [228]. This process serves as an easy and attractive decontamination strategy since it successfully destroys the microorganisms that cause food deterioration and hence reduces mycotoxins. The main benefit of γ radiation is that it can compromise the quality of food in respect of its nutritional values and sensory properties; however, the efficiency of this strategy is governed by various factors, such as type and amount of fungal lineages, radiation dose, food composition, and humidity of surroundings [228].

Different adsorbents and binders with a large specific surface area are used as decontaminating agents that can bind toxins, from contaminated feed, in the gastrointestinal tract of animals. Thus, these toxins can be eliminated through feces and their bioavailability and systemic absorption would be diminished [300,301]. Macroporous resins activated carbon and diatomite are the most commonly used physical adsorbents for the removal of mycotoxins. The interaction between the adsorbent and mycotoxin takes place through hydrophobic bonding, hydrogen bonds, coordination bonds, or electrostatic attraction/repulsion. However, this approach is incapable of degrading toxins and may decrease the nutrient content of the feed, thereby impacting its nutritional value and taste [243]. Up to now, no adsorbent has been approved by the EC for DON removal, probably due to the ineffectiveness of several materials, including clay-based minerals. For instance, Murugesan et al. [53] examined twenty-seven commonly used feed additives for binding mycotoxins and found that the absorption rates of DON were less than 25% in in vitro for all items. Recently, it was found that specific activated carbon can limit DON absorption in the GIT of pigs [302]. Although this approach is promising, it is only suitable for short-term usage since activated carbon is a nonspecific adsorbent that also binds and removes essential nutrients.

**Chemical Methods:** chemical methods for detoxification of type B trichothecenes include the application of various chemical agents, such as oxidizing agents (e.g., chlorine, moist and dry ozone, or sodium hypochlorite), reducing agents (e.g., sodium bisulfite and sodium metabisulfite), alkaline agents (e.g., ammonia, sodium, or calcium hydroxide), and other agents [88,182,243,303]. Ozone has a high oxidizing potential, which makes it a highly reactive gas. It has been successfully utilized to prevent mold growth and control different mycotoxins in food products since it is considered safe and environmentally friendly [175,304]. Recently, the ozonation process (62 mg/L ozone for 240 min) proved to be an efficient strategy in the detoxification of DON and ZEN in naturally contaminated wheat bran without influencing the total phenolic compound content and antioxidant activity of the wheat bran [304].

Cromey et al. [305] utilized tebuconazole chemical, a triazole fungicide, and succeeded to mitigate mycotoxin levels and FHB in cereal grains. Although most of the physical and chemical methods are economically affordable, they also have certain limitations. For instance, heat treatment processes that are performed at elevated temperatures can adversely affect the quality of raw food materials [306]. Rinsing and washing food materials leaves trichothecenes and their byproducts in solution, which may cause secondary contamination [307]. On the other hand, the use of chemicals such as acids, bases, aldehydes, and oxidizing agents for the decontamination of toxins can alter their structures, and result in the formation of degradation products with toxic chemical residues in foodstuffs and feed, thereby raising deleterious side effects and further public health issues [182,278]. Changing the grain’s nutritional value and quality is also another limitation associated with the application of chemical methods, which limits their application at a large scale [88]. For instance, ammonia or any powerful oxidizing chemicals can efficiently mitigate toxin contamination, and also decreases the nutritional quality of the foodstuffs at the same time [308]. As a result, chemical methods are somewhat restricted by manufacturers, while eco-friendly biological methods using microorganisms and enzymes have been commonly proposed as favorable and reliable alternative strategies for controlling mycotoxin contamination in foods.

**Biological Methods:** biological decontamination techniques involve the use of microorganisms (bacteria, fungi, yeast, actinomycetes, etc.) and enzymes to mitigate or remove type B trichothecenes from food materials using two main mechanisms; adsorption or enzymatic degradation [306]. Currently, biological degradation has become a hotspot issue on a global scale due to its high efficiency, specificity, minimal negative effects, and environmental friendliness. For biological decontamination of type B trichothecenes, microbial modification can be performed using various processes, such as acetylation, deacetylation, oxidation, glucosylation, de-epoxidation, and epimerization [309]. It is generally recognized that the modification products of trichothecene are less toxic for humans and animals compared to its parent form.

In recent times, it has been found that certain fungi are capable of degrading type B trichothecenes into less harmful compounds. An inspired work was conducted by He and coworkers for the isolation of a strain of *Aspergillus Tubingensis* (NJA-1) from soil samples by inorganic salt culture media supplemented with DON [310]. The NJA-1 showed high efficiency to hydrolyze DON with an average DON biotransformation rate of 94.4% after two weeks of cultivation, and thus managed to improve the state of DON poisoning in mice. Tian et al. [311] evaluated a biological control-based strategy for DON produced by *F. graminearum* 5035 in dual culture tests using eight selected *Trichoderma* strains. They found that *T. harzianum* Q710613, *T. atroviride* Q710251, and *T. asperellum* Q710682 are efficient to reduce DON content at high rates (more than 90% compared with the control). Interestingly, the authors also detected DON-3G when incubating *Trichoderma* with *F. graminearum*, which indicated that *Trichoderma* strains bound DON with glucose moiety to enable its transformation into a less toxic metabolite (i.e., masked mycotoxin) through a self-defense mechanism. Garda-buffon et al. [312] found that *Aspergillus oryzae* and *Rhizopus oryzae* were effective not only to degrade DON but also to enhance the activity of the peroxidase enzyme at the time interval when the greatest degradation of DON occurred.

Various bacteria have been explored for the detoxification of DON and other trichothecenes in food materials. The bacterial strain BBSH 797, an anaerobic bacterium isolated from rumen fluid, was successfully evaluated by EFSA for its safety and effectiveness for DON decontamination in pig feed additive, and accordingly, it was approved by the EC [313]. This bacterium (strain no. 11798) was assigned as a member of a *genus novus* of the family *Coriobacteriaceae*. In a recent study, lactic acid bacteria showed good efficiency in reducing the concentrations of DON and T2 in malting wheat by 58% and 34%, respectively [314]. Wang et al. [315] isolated *Bacillus licheniformis* strain YB9 from a moldy soil sample and found that it can degrade more than 82.7% of 1 mg/L DON within 48 h at 37 °C. Zhang et al. [316] reported that *Pelagibacterium halotolerans* ANSP101 from ocean sources can transform DON into a less toxic product, 3-keto-DON. Another novel bacterium (*Eggerthella sp. DII-9*) that was isolated from a chicken intestinal tract exhibited great efficiency in the detoxification of trichothecene mycotoxins (DON, T2, HT2, and T2 tetraol) [317]. Yeasts and other fermenting organisms are capable of binding certain trichothecenes from agricultural commodities onto their cell wall surface constituents (e.g., glucomannans, β-D-glucans, mannan oligosaccharides), thus efficiently decontaminating food and feed. Garda et al. [318] tested the alcoholic fermentation of malt with *Saccharomyces cerevisiae* and succeeded in reaching a total decontamination rate of 53% for DON and T-2 toxin. Various studies have shown that biological detoxification methods are efficient and cost-effective with minor effects on the nutritional value of food; however, the applicability of biological control agents has been commonly investigated in in vitro experiments or at the laboratory scale [175]. Therefore, their uses on mycotoxins should be extended to large-scale detoxification applications. On the other hand, various bioactive compounds, such as phytochemicals and their essential oils (cinnamon oil, propyl paraben, butylated hydroxy anisole, and clove essential oils) have been recently explored as natural, safe, and eco-friendly preservatives with antimicrobial properties for inhibition of trichothecenes production and enhancing the shelf life of foodstuffs [274]. Other innovative technologies based on photocatalytic degradation and electrochemical oxidation have been also established for easy, effective, eco-friendly, and inexpensive degradation or elimination of various mycotoxins, including type B trichothecenes in food materials [88].

## 9. Conclusions and Future Outlook

Due to the widespread geographical occurrence of type B trichothecenes and DON-3G in cereal grains, as well as the increasing concerns regarding their health impacts, an in-depth understanding of various aspects related to their toxicology, distribution, quantitative analysis, and management strategies is of paramount importance. Acute and chronic exposure to type B trichothecenes can elicit a variety of adverse health effects, such as food refusal, vomiting, weight loss, neural disturbances, gastrointestinal discomfort, hematological disorders, inhibition of protein synthesis, and immunosuppression. Trichothecene-generating *Fusarium* fungi colonize crops at the pre-harvest stages of production, which makes it difficult to avoid their contamination. Type B trichothecenes and DON-3G have been found in various cereal grains, especially in wheat, maize, barley, and oats. Their concentrations may vary due to complex interactions of biotic and abiotic conditions, geographical locations, storage management, etc. More research in this area is still needed to better understand the impacts of mycotoxin co-contamination and their potential synergism.

Currently, SPE, IACs, and MFCs are the preferred clean-up procedures to retain and purify type B trichothecenes from various food matrices. A variety of detection techniques have been established for type B trichothecenes. Among them, LC-MS/MS is the most popular technique due to its high separation capacity, outstanding detection sensitivity, fast acquisition features, and compatibility with a broad range of sample preparation procedures. However, it has been observed that researchers are migrating toward biosensor-based approaches due to their ease of use, rapidity, low cost, high sensitivity, and selectivity. Even still, some conjugated forms of mycotoxins are still unnoticed and are hardly detected by routine determination, thereby inducing a serious hazard to food safety. Therefore, further studies on conjugated trichothecenes for easy detection and control will necessitate more comprehensive and in-depth research. Considering the continuous evolution of analytical techniques, it is anticipated that conventional chromatographic techniques will be replaced by HRMS, such as quadrupole Orbitrap and time of flight, which are particularly useful for the identification and non-targeted detection of metabolites or other forms of conjugated mycotoxins.

From the perspective of toxin control technology, various pre-harvest and post-harvest strategies have been employed to minimize the growth of *Fusarium* toxigenic fungi in field crops and to detoxify type B trichothecene in contaminated foods. GAP measures such as appropriate crop rotation, soil tillage, variety selection, planting date, and production of genetically engineered plants are the most reliable approaches to hindering the growth of the toxigenic fungi in the farm. For post-harvest management strategies, appropriate grain drying, sorting, and storage under controlled environmental conditions are important measures to control the development of mycotoxins in grains. Although various decontamination techniques relying on physical and chemical methods have been proposed, most of them are not technically feasible. The growing awareness of the ecological effects of synthetic chemicals has focused the interest of researchers on using more advanced and eco-friendly biotechnology approaches based on gene silencing through RNA interference (RNAi) and protein engineering, which may offer a promising perspective for the biological control of type B trichothecenes in various food materials. In addition, the use of phytochemicals and their essential oils have been proven natural, safe, and eco-friendly for type B trichothecene detoxification. Ultimately, the availability of absolute measures for the removal of these mycotoxins from the food chain is still missing; however, potential hazards associated with their presence in food can be reduced by a combination of efficient pre-harvest, harvest, and post-harvest strategies together with appropriate quality assurance programs.

## Figures and Tables

**Table 2:** Representative occurrence studies on type B trichothecenes in different food commodities around the world.

Cereal Grain	Year of Study	Detection Technique	Number of Samples	Type B Trichothecene	Incidence (%)	Mean (µg/kg)	Range (µg/kg)	Country	Reference
Wheat flour	2012–2013	UPLC-MS/MS	672	DON	91.5	178	2.4–1130	China	[95]
				3-ADON	1.64	2.1	1.5–2.6		
				15-ADON	18.0	1.3	0.62–6.0		
Wheat	2018	LC-MS/MS	338	DON	90.8	2628	2.0–59278	China	[140]
				3-ADON	69.2	135	28.8–13409		
				15-ADON	49.4	50.2	27.0–805		
				NIV	45.3	266	13.1–3044		
Wheat	2016	HPLC-UV	92	DON	70.0	140	5.1–373	Poland	[97]
				NIV	83.0	35.0	10.5–1265		
				DON-3G	27.0	41.9	15.8–138		
Wheat flour	2019–2020	UPLC-MS/MS	27	DON	96.0	38.1	0.74–154	Republic of Korea	[26]
				DON-3G	96.0	6.8	0.25–24.7		
				NIV	85.0	23.7	0.45–126		
				3-ADON	11.0	6.34	2.3–10.3		
				15-ADON	18.5	14.3	6.0–30.6		
				FUS-X	37.0	2.4	0.80–4.6		
Wheat flour	2020	UPLC-PDA	50	DON	56.0	188	<LOQ–389	Egypt	[102]
				NIV	34.0	100	<LOQ–179		
				DON-3G	24.0	84	<LOQ–120		
Wheat	2012–2014	LC-MS/MS	84	DON	100.0	1762	<LOQ–9480	Argentina	[98]
				3/15-ADON	49.0	52	<LOQ–190		
				DON-3G	93.0	198	<LOQ–850		
Wheat	2015–2018	HPLC-PDA	50	DON	26.0	433	58.0–1092	Turkey	[123]
Wheat flour			50		6.0	116	92.0–151		
Wheat bread			60		ND	-	-		
Wheat (summer)	2018–2019	HPLC-UV	232	DON	44.8	1051	<LOQ–2145	Pakistan	[99]
Wheat (winter)			217		41.9	840	<LOQ–2050		
Wheat bran	2013	HPLC-UV	37	DON	62.0	1308	NA–6178	Spain	[141]
Wheat	2013	LC-MS/MS	57	DON	16.0	11.0	9.6–99.6	Italy	[83]
				3-ADON	ND	-	-		
				15-ADON	2.0	0.64	10.8–29.1		
				FUS-X	14.0	18.4	12.5–102		
				NIV	11.0	8.9	12.0–106		
Wheat	2015	HPLC-MS/MS	368	DON	100.0	17754	109.6–86,255	China	[96]
				DON-3G	99.5	414	28.3–2957		
				NIV	87.8	250	0.60–2399		
				3-ADON	80.0	39.6	0.60–284		
				15-ADON	67.3	13.2	3.0–185		
				FUS-X	35.2	8.3	1.8–48.2		
Wheat	2015	LC-MS/MS	30	3-ADON	46.7	24.6	NA–71.0	Finland	[14]
				DON	96.7	866	NA–5510		
				DON-3G	83.3	174	NA–922		
				NIV	43.3	48.9	NA–74.0		
Wheat	2014	HPLC-MS/MS	47	DON	59.6	172	13.0–1230	Italy	[142]
				3-ADON	8.5	23.0	4.0–33.0		
				15-ADON	32.0	45.0	12.0–105		
				NIV	6.3	183	67.0–290		
				FUS-X	6.3	8.0	5.0–14.0		
Wheat	2014	HPLC-MS/MS	40	DON	22.5	172	9.0-550	Syria	[142]
Wheat	2012	LC-MS/MS	20	DON	50.0	-	22.8–113	Malaysia	[119]
Wheat	2010	LC-MS/MS	41	DON	100.0	1075	152–2550	China	[93]
	2011		64		32.8	82.1	14.5–1580		
	2012		75		96.0	307	16.3–41157		
Wheat	2014	LC-MS/MS	80	DON	5.0	653	NA–1480	Morocco	[143]
Wheat	2009	LC-MS/MS	23	DON	100.0	1500	203–4130	Austria, Germany, and Slovakia	[101]
				DON-3G	100.0	393	76.0–1070		
Wheat	2017	UPLC-MS/MS	579	DON	100.0	166	12.2–6436	China	[144]
				3-ADON	4.2	1.2	<LOQ–150		
				15-ADON	0.52	0.20	<LOQ–24.5		
Wheat	2005–2007	UPLC-PDA	55	DON	34.5	190	57.0–423	Serbia	[105]
Wheat	2016	LC-MS/MS	150	DON	98.0	-	183–2150	Brazil	[145]
Wheat biscuits	2010	LC-MS	201	NIV	41.0	3.1	1.4–35.0	Japan	[146]
				DON	98.0	23.0	0.90–791		
Wheat	2007–2010	LC-MS/MS	686	DON	84.0	647	NA–10,600	Switzerland	[147]
				NIV	19.0	15.0	NA–470		
Wheat	2013	HPLC-UV	50	DON	40.0	910	7.0–4730	India	[110]
Wheat	2013	ELISA/HPLC	51	DON	65.0	223	115–278	Croatia	[111]
Corn (winter)	2018–2019	HPLC-UV	142	DON	61.2	1122	<LOQ–2967	Pakistan	[99]
Corn (summer)			128		44.5	817	<LOQ–2490		
Maize	2010	ELISA/TLC and ELISA/HPLC	40	DON	85.0	2150	15.0–17,900	Croatia	[100]
Maize	2008	LC-MS/MS	54	DON	100.0	753	238–3680	Austria, Germany, and Slovakia	[101]
				DON-3G	100.0	141	25.0–763		
Corn	2011	GC–ECD	25	NIV	52.0	25.4	NA–129	Republic of Korea	[84]
				DON	96.0	110	NA–492		
				FUS-X	24.0	2.1	NA–19.1		
				15-ADON	80.0	17.3	NA–98.0		
				3-ADON	28.0	1.1	NA–6.8		
Corn flour	2019–2020	UPLC-MS/MS	25	DON	96.0	441	0.79–1223	Republic of Korea	[26]
				DON-3G	96.0	99.7	0.14–419		
				NIV	80.0	57.5	1.9–234		
				3-ADON	72.0	4.6	1.2–11.8		
				15-ADON	60.0	135	38.6–298		
				FUS-X	60.0	13.0	1.2–28.7		
Corn flour	2020	UPLC-PDA	45	DON	83.3	330	<LOQ–853	Egypt	[102]
				NIV	74.1	147	<LOQ–462		
				DON-3G	40.7	121	<LOQ–257		
Maize	2017	LC-MS/MS	79	DON	8.0	311	NA–807	Egypt	[148]
				DON-3G	5.0	-	NA–47.5		
				NIV	10.0	-	NA–38.6		
Maize	2007	HPLC-MS	180	DON	22.2	226	9.6–745	Southwest Nigeria	[103]
				3-ADON	17.2	17.0	0.70–72.4		
Maize	2015	HPLC-MS	39	DON	70.7	0.30	0.10–0.70	Northeast Nigeria	[104]
Maize	2003–2005	HPLC-MS	312	DON	70.0	1104	NA–10626	Asia and Oceania	[149]
			244		80.0	1073	NA–3970	Europe and the Mediterranean region	
Maize	2017	UPLC-MS/MS	606	DON	99.8	175	<LOQ–4300	China	[144]
				3-ADON	13.5	5.0	<LOQ–385		
				15-ADON	76.4	115	<LOQ–4811		
Maize	2015–2018	UPLC-MS/MS	15	DON	13.3	322	313–331	Turkey	[123]
Corn-based food	2008	GC-ECD	175	DON	26.8	53.9 (median)	26.1–132	Spain	[150]
				NIV	4.0	60.2 (median)	51.1–107		
Maize	2013	LC-MS/MS	37	DON	100.0	171	43.0–435	Cameroon	[151]
				DON-3G	100.0	16.0	<LOQ–82.0		
				NIV	100.0	161	3.0–782		
				FUS-X	86.0	43.0	<LOQ–112		
Corn	2014	LC-MS/MS	10	DON	90.0	627	74.0–1382	China	[19]
				NIV	40.0	25.0	19.0–53.0		
				DON-3G	80.0		14.0–121		
				FUS-X	ND	-	-		
				3-ADON	ND	-	-		
				15-ADON	60.0	40.0	15.0–79.0		
Corn	2016	HPLC-UV	44	DON	66.0	-	5.8–9843	China	[152]
Maize	2005–2007	UPLC-PDA, ELISA	216	DON	32.4	223	27.0–2210	Serbia	[105]
Maize	2009–2011	LC-MS/MS	140	DON	21.4	-	3.0–428	Italy	[106]
Maize	2015	LC-MS/MS	60	DON	63.0	490	68.0–2196	Tanzania	[107]
Maize	2015	HPLC-PDA	30	DON	66.6	50.8	<LOQ–90.0	Poland	[108]
Maize	2016	LC-MS/MS	29	DON	86.0	1872	225.0–2963	Hungary	[109]
Maize	2013	HPLC-UV	25	DON	24.0	450	10.0–1070	India	[110]
Maize	2013	ELISA/HPLC	63	DON	71.0	1565	215–2942	Croatia	[111]
Oats	2015	LC-MS/MS	31	3-ADON	77.4	341	NA–2720	Finland	[14]
				DON	100.0	2690	NA–23,800		
				DON-3G	87.1	806	NA–6600		
				NIV	71.1	635	NA–4940		
Oat kernel	2015–2019	LC-MS/MS	100	DON	22.0	81.4	19.1–736	Spain	[112]
				3-ADON	3.0	29.9	9.2–42.6		
Oat bran	2013	LC-MS/MS	30	DON	17.0	230	NA–276	Spain	[141]
Oat	2013–2015	LC-MS/MS	325	DON	49.0	56.9	NA–1328	Switzerland	[113]
				NIV	64.3	108	NA–1653		
Oat flakes		LC-MS/MS	25	DON	60.0	44.0	16.8–244	China	[114]
Oat	2016–2018	UHPLC-HRMS	168	DON	55.3	475	NA–4143	Canada	[115]
				NIV	92.0	210	NA–795		
Oat	2013	LC-MS/MS	7	DON	57.0	29.9	10.3–83.0	Italy	[83]
				3-ADON	14.2	0.74	<LOQ–5.23		
				15-ADON	ND	-	-		
				FUS-X	42.8	23.0	26.0–75.0		
				NIV	57.0	27.1	45.5–50.4		
Oat	2017–2018	HPLC-UV	11	DON	ND	-	-	Republic of Korea	[116]
				DON-3G	ND	-	-		
				NIV	9.1	23.5	23.5		
Oat	2010–2011	LC-MS/MS	93	DON	95.0	-	99.0–5544	Sweden	[117]
				NIV	91.5	-	18.0–1743		
Oat	2006–2008	LC-MS/MS	303	NIV	73.0	57.0	NA–741	UK	[118]
				FUS-X	1.0	<10.0	NA–18.0		
				DON	32.0	28.0	NA–1866		
Oat	2013	LC-MS/MS	10	DON	30.0	-	22.7–100	Malaysia	[119]
Oat	2005–2010	HPLC-UV	52	DON	30.0	170	NA–490	Slovakia	[120]
Oat	2005–2010	HPLC-UV	52	DON	4.0	90.0	90.0–94.0	Russia	[153]
Oat	2004–2009	LC-MS/MS	289	DON	90.0	-	NA–30,000	Norway	[154]
				3-ADON	71.0	-	NA–5100		
				NIV	1.0	-	NA–310		
				FUS-X	ND	-	-		
Oat	2013	ELISA/HPLC	33	DON	21.0	145	34.0–201	Croatia	[111]
Barley	2015	LC-MS/MS	34	3-ADON	41.2	-	NA–18.3	Finland	[14]
				DON	82.4	234	NA–802		
				DON-3G	73.5	148	NA–594		
				NIV	73.5	96.6	NA–262		
Barley	2009	HPLC-UV	70	DON	54.0	9.4	3.7–36.8	Republic of Korea	[121]
Barley	2011	GC-ECD	39	NIV	59.0	16.6	NA–101	Republic of Korea	[84]
				DON	56.0	16.4	NA–40.1		
				FUS-X	15.0	1.0	NA–9.9		
				15-ADON	31.0	1.1	NA–7.1		
				3-ADON	26.0	0.70	NA–3.9		
Barley	2020	HPLC-UV	15	DON	33.3	75.8	11.7–286	Republic of Korea	[116]
				DON-3G	13.3	19.3	18.0–20.6		
				NIV	40.0	90.2	17.3–230		
Barley	2013	LC-MS/MS	9	DON	11.0	3.9	<LOQ–35.5	Italy	[83]
				3-ADON	ND	-	-		
				15-ADON	ND	-	-		
				FUS-X	44.4	18.4	27.5–47.3		
				NIV	33.3	25.2	21.7–106		
Barley	2012	LC-MS/MS	10	DON	50.0	-	27.9–72.5	Malaysia	[119]
Barley beer	2018	LC-MS/MS	100	NIV	56.0	2.4 µg/L	0.50–7.6 µg/L	Poland	[122]
				DON	83.0	9.0 µg/L	1.0–73.6 µg/L		
				DON-3G	67.0	9.2 µg/L	2.0–35.8 µg/L		
Barley	2016	LC-MS/MS	36	DON	48.0	-	240–429	Hungary	[109]
Barley	2013	HPLC-UV	25	DON	16.0	210	30.0–530	India	[110]
Barley	2018	LC-MS/MS	76	DON	94.0	5000	310–15500	Brazil	[85]
Barley	2013	ELISA/HPLC	34	DON	53.0	342	74.0–228	Croatia	[111]
Barley	2009	HPLC-UV	72	DON	56.9	400–2200	500–3600	Tunisia	[155]
Barley	2020	HPLC-PDA	15	DON	20.0	425	138–973	Turkey	[123]
Barley	1997–2000	HPLC-PDA	93	DON	9.0	2553	1470–4000	Saudi Arabia	[156]
				NIV	1.0	3.1	3.1		
Rice	2020	HPLC-PDA	20	DON	35.0	195	136–256	Turkey	[123]
Rice	2018	LC-MS/MS	180	NIV	28.0	13.8	<LOQ–116	Pakistan	[124]
				DON	8.0	6.9	<LOQ–115		
White rice	2018	HPLC-UV	241	NIV	21.0	33.5	12.6–2175	Republic of Korea	[125]
				DON	5.0	4.0	7.1–372		
Brown rice	2018	HPLC-UV	241	NIV	34.0	52.2	17.3–2534		
				DON	7.0	5.4	9.1–435		
Rice	2011	GC-ECD	65	NIV	35.0	9.5	NA–45.0	Republic of Korea	[84]
				DON	15.0	3.7	NA–31.7		
				FUS-X	15.0	1.4	NA–15.0		
				15-ADON	46.0	2.0	NA–24.1		
				3-ADON	12.0	0.60	NA–10.0		
Glutinous rice	2011	GC-ECD	11	NIV	64.0	11.9	NA–23.1	Republic of Korea	[84]
				DON	10.0	1.7	NA–18.2		
				FUS-X	18.0	1.0	NA–8.9		
				15-ADON	45.0	1.3	NA–4.2		
				3-ADON	ND	-	-		
Brown rice	2011	GC-ECD	48	NIV	60.0	14.5	NA–45.4	Republic of Korea	[84]
				DON	33.0	7.1	NA–24.9		
				FUS-X	2.0	0.40	NA–18.7		
				15-ADON	25.0	0.5	NA–3.1		
				3-ADON	29.0	1.5	NA–10.2		
Rice	2014	GC-MS/MS	23	DON	13.0	5.0	NA–5.5	Spain	[126]
				3-ADON	ND	-	-		
				FUS-X	ND	-	-		
				NIV	ND	-	-		
Rice	2015	LC-MS	41	DON	70.7	0.30	0.10–0.7	Nigeria	[104]
Rice	2011	HPLC-UV	21	DON	23.8	18.9	11.2–112	Nigeria	[157]
Rice	2012	LC-MS/MS	50	DON	26.0	-	12.5–81.2	Malaysia	[119]
Rice flour	2014–2016	LC-MS/MS	93	DON	5.3	33.0	NA–125	Brazil	[127]
				ADON	73.3	3.8	NA–17.0		
Rice-based cereals	2008	GC-MS	29	DON	3.4	1.4	-	Canada	[158]
Sorghum	2017–2018	UPLC-UV		DON	33.3	81.7	30.0–159	Republic of Korea	[159]
				NIV	46.7	97.6	29.0–206		
Sorghum	2017–2018	HPLC-UV	12	DON	100.0	119	18.9–712	Republic of Korea	[116]
				DON-3G	41.7	18.8	10.4–43.4		
				NIV	91.7	45.3	4.6–146		
Sorghum	2018	LC-MS/MS	110	DON	3.0	100	NA–119	Nigeria	[128]
				15-ADON	2.0	39.0	NA–44.0		
				DON-3G	23.0	24.0	NA–63.0		
				NIV	ND	-	-		
				FUS-X	ND	-	-		
Sorghum	2021	LC-MS/MS	20	DON	ND	-	-	Nigeria	[129]
				3-ADON	ND	-	-		
				15-ADON	ND	-	-		
Sorghum	2018	LC-MS/MS	1533	DON	0.46	63.3	40.0–112	Sub-Saharan countries	[130]
Sorghum	1999	HPLC-UV	33	DON	90.9	360	50.0–2340	Ethiopia	[131]
				NIV	9.1	307	50.0–490		
Sorghum	2019	LC-MS/MS	12	NIV	17.0	49.5	47.6–51.4	Togo	[132]
				DON	17.0	25.9	19.0–32.8		
Sorghum	2012	LC-MS/MS	4	DON	ND	-	-	Tunisia	[133]
				NIV	100.0	-	418–667		
Rye	2013	LC-MS/MS	11	DON	45.4	23.2	16.5–79.6	Italy	[83]
				3-ADON	ND	-	-		
				15-ADON	ND	-	-		
				FUS-X	45.4	28.5	42.4–70.2		
				NIV	27.3	56.9	33.9–34.4		
Rye	2010	LC-MS/MS	15	DON	100.0	270	87.0–500	Canada	[135]
				ADONs	ND	-	-		
Rye	2009	LC-MS/MS	61	DON	100.0	28	NA–288	Germany	[136]
				3-ADON	59.0	0.39	NA–5.0		
				15-ADON	80.0	0.73	NA–8.6		
				NIV	3.3	0.06	NA–1.8		
				FUS-X	1.6	0.01	NA–1.8		
Rye	1998–2001	GC-ECD	69	DON	59.4	42.5	NA–257	Denmark	[138]
				NIV	13.0	11.5	NA–48.0		
Rye	2014 and 2015	GC-ECD	117	DON	75.0	1060	<LOQ–10760	USA	[139]
Conventional rye	2009–2012	LC-MS/MS	18	NIV	ND	-	-	Poland	[160]
				DON	83.0	29.5	NA–120		
				3-ADON	ND	-	-		
Organic rye	2009–2012	LC-MS/MS	23	NIV	4.0	-	<20	Poland	[160]
				DON	26.0	<15	NA–41.6		
				3-ADON	ND	-	-		

Abbreviation: DON: deoxynivalenol; NIV: nivalenol; 3-ADON: 3-acetyldeoxynivalenol; 15-ADON: 15-acetyldeoxynivalenol; FUS-X: fusarenon-X; DON-3G: deoxynivalenol-3-glucoside; UPLC-MS/MS: ultra-performance liquid chromatography–tandem mass spectrometry; HPLC-UV: high-performance liquid chromatography coupled with ultraviolet detection; HPLC-PDA: high-performance liquid chromatography coupled with photodiode array detection; ELISA: enzyme-linked immunosorbent assay; TLC: thin layer chromatography; LC-MS: liquid chromatography-mass spectrometry; LC-MS/MS: liquid chromatography–tandem mass spectrometry; GC-ECD: gas chromatography–electron capture detection; GC-MS: gas chromatography–mass spectrometry; GC-MS/MS: gas chromatography–tandem mass spectrometry; ND: not detected; NA: not available in publication.

**Table 3:** Advantages and disadvantages of analytical methods for type B trichothecenes.

Method	Advantages	Limitations	Reference
Thin layer chromatography	Simple and rapid Low-cost separation technique Reliable quantification method when combined with densitometry	Outdated technique Poor precision and sensitivity Destructive sample preparation Quantitative only when combined with a densitometer Largely substituted by high-performance liquid chromatography for quantitative determination of trichothecenes Inherent need for sample preparation	[2,161,166]
High-performance liquid chromatography (HPLC)	Good sensitivity, selectivity, and repeatability Automated Short analysis time Official reference method for the validation and verification of immunochemical tests	Destructive sample preparation Expensive technique Requires dedicated operator Derivatization may be required	[175,243]
Liquid chromatography/mass spectrometry	High selectivity and repeatability Very low detection limits (LC-MS/MS) Fast acquisition features Compatibility with a broad range of sample preparation procedures Wide linear dynamic range Simultaneous determination of numerous mycotoxins Ability to generate structural information of analyte (HRMS) No derivatization required Minimum requirement for sample preparation (LC-MS/MS)	Destructive sample preparation Very expensive technique Requires dedicated operator and specialist expertise for data interpretation Sensitivity relies on the ionization method	[8,18,167]
Gas chromatography	Good separation ability and repeatability Very low detection limits (GC-MS/MS) Automated Simultaneous analyses of multiple mycotoxins	Expensive technique Requires dedicated operator Matrix interferences Requires derivatization for nonvolatile mycotoxins Carry-over effects from previous samples Narrow scope of analysis	[236,237,241]
Enzyme-linked immunosorbent assay (ELISA)	Inexpensive and specific assay Reduced analysis time Visual assessment Easy manipulation Semi-quantitative (screening) or quantitative analysis is possible No dedicated operator required Limited consumption of organic solvents	Easily affected by matrix interferences Affected by potential cross-reactivity with structurally related toxins One-time use only Inefficiency in detection at low concentrations Semiquantitative Confirmatory LC analysis is often required Possible false positives/negatives Narrow detection range	[175,246,274]
Lateral flow immunochromatographic assay	Rapid and straightforward (single-step) test No special equipment required Inexpensive onsite screening test No additional chemicals or laborious preparation processes required Portable Reliable quantification method when combined with other modern technology	Semiquantitative (visual assessment) Affected by potential cross-reactivity with structurally related toxins Requiring validation for additional matrices	[255,272,275]
Fluorescence polarization immunoassay	Mobility due to portable instrumentation Very sensitive, rapid and user-friendly Homogeneous method performed in the solution phase Faster detection with no additional clean-up and washing steps Convenient for monitoring large-scale samples	Possible cross-reactivity with structurally related toxins Limited validation with HPLC or ELISA Matrix interferences Limited to a single mycotoxin detection at a time	[257,258,272]
Biosensors	High transmission and low-cost operation High sensitivity and selectivity User-friendly operation Reduced analysis time Mobility due to portable instrumentation Ability to be recycled Self-contained, simple design	Extensive sample preparation is required to improve sensitivity Limited to a single mycotoxin detection at a time Possible cross-reactivity with structurally related toxins Variable repeatability and reproducibility (enhanced when using novel materials)	[260,261,272]
Near-infrared spectroscopy	Reduced analysis time Easy operation Non-destructive testing with minimal or no sample manipulation Quick classification of grains according to mycotoxin contamination	Reliable only when combined with appropriate mathematical tools such as principal component analysis Complicated interpretation of spectral data Knowledge of statistical methods is required Validation of the calibration model is required Expensive equipment Poor sensitivity (high limit of detection) Point-based scanning method which enables only a mean spectrum (average measurement)	[167,264,268]
Hyperspectral imaging	Reduced analysis time Easy operation Non-destructive testing with minimal or no sample manipulation Information about the spatial distribution of chemical constituents across the sample is provided (sample heterogeneity can be overcome) High spectral and spatial resolution Quick classification of grains according to mycotoxin contamination	Reliable only when combined with appropriate mathematical tools such as principal component analysis Complicated interpretation of spectral data Knowledge of statistical models is required Validation of the calibration model is required Expensive equipment Poor sensitivity (high limit of detection)	[265,266,267]
Electronic nose (EN)	Rapid, inexpensive, and user-friendly screening method to distinguish the microbiological quality of food samples.	Enhancing selectivity and sensitivity is required Reducing interferences (e.g., to humidity) is required Nonvolatile mycotoxins raise difficulties for EN-based detection. Compensation for drift effects is required Narrow scope of analysis and poor validation	[163,269,270]
Capillary electrophoresis	Rapid analysis Convenient for separating closely related toxins Limited consumption of organic solvents Good selectivity of analytes from interferences Good sensitivity	Destructive sample preparation Limited to lab use due to cumbersome instrumentation Extensive sample preparation is required to improve sensitivity	[163,272]

Abbreviation: LC-MS/MS: liquid chromatography–tandem mass spectrometry; HRMS: high-resolution mass spectrometry, GC-MS/MS: gas chromatography–tandem mass spectrometry.

## Data Availability

Not applicable.

## Abbreviations

The following abbreviations are used in this manuscript:

15-ADON	15-Acetyldeoxynivalenol
3-ADON	3-Acetyldeoxynivalenol
AFs	Aflatoxins
ARfD	Acute reference dose
ASE	Accelerated solvent extraction
CAs	Chromosomal aberrations
CE	Capillary electrophoresis
CEN	European Committee of Standardization
DON-3G	Deoxynivalenol-3-glucoside
DNA	Deoxyribonucleic acid
DON	Deoxynivalenol
DSPE	Dispersive solid-phase extraction
dsRNA	Double-stranded RNA
EC	European Commission
ECD	Electron capture detection
EFSA	European Food Safety Authority
EN	Electronic nose
ELIZA	Enzyme-linked immunosorbent assay
EU	European Union
FAO	Food and Agriculture Organization
FHB	*Fusarium* head blight
FID	Flame ionization detection
FPIA	Fluorescence polarization immunoassay
FTNIR	Fourier transform NIR
FUS-X	Fusarenon-X
GAPs	Good agricultural practices
GC	Gas chromatography
GCB	Graphitized carbon black
GC-MS	Gas chromatography–mass spectrometry
GC-MS/MS	Gas chromatography–tandem mass spectrometry
GHPs	Good hygiene practices
GMPs	Good management practices
GSPs	Good storage practices
Hck	Hematopoietic cell kinase
HLB	Hydrophilic–lipophilic balanced
HPLC-PDA	High-performance liquid chromatography–photodiode array detection
HPLC-UV	High-performance liquid chromatography–ultraviolet detection
HRMS	High-resolution mass spectrometry
HS-GC/MS	Headspace–gas chromatography/mass spectrometry
HT2	HT-2 toxin
IACs	Immunoaffinity columns
IARC	International Agency for Research on Cancer
IECs	Intestinal epithelial cells
JECFA	Joint FAO/WHO Expert Committee on Food Additives
LC	Liquid chromatography
LC-FLD	Liquid chromatography–fluorescence detection
LC-MS	Liquid chromatography–mass spectrometry
LC-MS/MS	Liquid chromatography–tandem mass spectrometry
LFIA	Lateral flow immunochromatographic assay
LLE	Liquid–liquid extraction
LOD	Limit of detection
LOQ	Limit of quantification
mAb	Monoclonal antibody
MAE	Microwave-assisted extraction
MAPKs	Mitogen-activated protein kinases
MOS	Metal oxide semiconductors
MFC	Multifunctional clean-up columns
MIR	Mid-infrared spectroscopy
MOF	Metallic organic framework
MRLs	Maximum regulatory limits
mRNA	Messenger ribonucleic acid
MS	Mass spectrometry
MWFPIA	Multi-wavelength fluorescence polarization immunoassay
NA	Not available
ND	Not detected
NIR	Near-infrared spectroscopy
NIR-HIS	Near-infrared spectroscopy–hyperspectral imaging
NIV	Nivalenol
PCA	Principal component analysis
PLE	Pressurized liquid extraction
PLS-DA	Partial least squares discriminant analysis
PMTDI	Provisional maximum tolerable daily intake
PRRSV	Porcine reproductive and respiratory syndrome virus
PSA	Primary secondary amine
PTC	Peptidyl transferase center
QuEChERS	Quick, easy, cheap, effective, rugged, and safe
RNA	Ribonucleic acid
RSD	Relative standard deviation
SCF	Scientific Committee on Food
SFE	Supercritical fluid extraction
SLE	Solid–liquid extraction
SPE	Solid-phase extraction
T2	T-2 toxin
TDI	Tolerable daily intake
TJP	Tight junction proteins
TLC	Thin layer chromatography
UPLC	Ultra-performance liquid chromatography
UPLC-MS/MS	Ultra-performance liquid chromatography–tandem mass spectrometry
WHO	World Health Organization
ZEN	Zearalenone
